# Evaluation of Gene-Based Family-Based Methods to Detect Novel Genes Associated With Familial Late Onset Alzheimer Disease

**DOI:** 10.3389/fnins.2018.00209

**Published:** 2018-04-04

**Authors:** Maria V. Fernández, John Budde, Jorge L. Del-Aguila, Laura Ibañez, Yuetiva Deming, Oscar Harari, Joanne Norton, John C. Morris, Alison M. Goate, NIA-LOAD family study group, NCRAD, Carlos Cruchaga

**Affiliations:** ^1^Department of Psychiatry, Washington University School of Medicine, St. Louis, MO, United States; ^2^Hope Center for Neurological Disorders, Washington University School of Medicine, St. Louis, MO, United States; ^3^Knight Alzheimer's Disease Research Center, Washington University School of Medicine, St. Louis, MO, United States; ^4^Department of Neuroscience, Ronald M. Loeb Center for Alzheimer's Disease, Icahn School of Medicine at Mount Sinai, New York, NY, United States

**Keywords:** gene-based, family-based, clustering, variance-component, transmission disequilibrium, rare variants, whole exome sequencing, Alzheimer's disease

## Abstract

Gene-based tests to study the combined effect of rare variants on a particular phenotype have been widely developed for case-control studies, but their evolution and adaptation for family-based studies, especially studies of complex incomplete families, has been slower. In this study, we have performed a practical examination of all the latest gene-based methods available for family-based study designs using both simulated and real datasets. We examined the performance of several collapsing, variance-component, and transmission disequilibrium tests across eight different software packages and 22 models utilizing a cohort of 285 families (*N* = 1,235) with late-onset Alzheimer disease (LOAD). After a thorough examination of each of these tests, we propose a methodological approach to identify, with high confidence, genes associated with the tested phenotype and we provide recommendations to select the best software and model for family-based gene-based analyses. Additionally, in our dataset, we identified *PTK2B*, a GWAS candidate gene for sporadic AD, along with six novel genes (*CHRD, CLCN2, HDLBP, CPAMD8, NLRP9*, and *MAS1L*) as candidate genes for familial LOAD.

## Introduction

Alzheimer disease (AD) is a complex condition for which almost 50% of its phenotypic variability is due to genetic causes; yet only 30% of the genetic variability is explained by known markers (Ridge et al., 2016). GWAS studies have identified more than 20 risk loci (Lambert et al., 2013) and sequencing studies have identified additional genes harboring low frequency variants with large effect size (*TREM2, PDL3, UNC5C, SORL1*, and *ABCA7*; Sims et al., 2017). Recent studies also indicate that Late-Onset AD (LOAD) families are enriched for genetic risk factors (Cruchaga et al., 2017). Therefore, studying those families may lead to the identification of novel variants and genes (Guerreiro et al., 2013; Cruchaga et al., 2014).

Current consensus is that the missing heritability for complex traits like AD may be hidden within rare variants that have low to moderate effect on disease risk (Frazer et al., 2009; Manolio et al., 2009; Cirulli and Goldstein, 2010). The rarity of these markers requires specific study designs and statistical analyses for their detection. The simplest approach to detect rare variants for association is to test each variant individually using standard contingency table and regression methods. But due to the limited number of observations of the rare minor allele for a specific variant, the statistical power to detect association with any rare variant is limited; hence, extremely large samples are required and a more stringent multiple-test correction is necessary (Li and Leal, 2008; Bansal et al., 2010). It has been acknowledged that the best alternative to single-variant analysis is to collapse sets of pre-defined candidate rare variants within significant units, usually genes (gene-based sets) (Neale and Sham, 2004; Lee et al., 2014). For collapsing tests each variant is given a certain weight and the weights of all variants within the region are summed; depending on the weights and how summation is performed there are three major types of gene-based methods: collapsing tests, variance-component tests, and combined tests (Lee et al., 2014). Collapsing tests analyze whether the overall burden of rare variants is significantly different between cases and controls by regressing disease status on minor allele counts (MAC). The Cohort Allelic Sum Test (CAST) is a dominant genetic model which assumes that the presence of any rare variant increases disease risk (Morgenthaler and Thilly, 2007); whereas the Combined Multivariate and Collapsing (CMC) method collapses rare variants in different MAF categories and evaluates the joint effect of common and rare variants through Hoteling's test (Li and Leal, 2008). However, neither CAST nor CMC tests account for directional effect. The Variable Threshold (VT) test does allow for both trait-increasing and trait-decreasing variants; it selects optimal frequency thresholds for burden tests of rare variants and estimates *p*-values analytically or by permutation (Price et al., 2010). Variance-componence methods test for association by evaluating the distribution of genetic effects for a group of variants while appropriately weighting the contribution of each variant. The sequence kernel association test (SKAT) casts the problem to mixed models (Lee et al., 2014) and, in the absence of covariates, SKAT reduces to a C-alpha test (Neale et al., 2011). Finally, collapsing and variance component tests can be combined into one statistical method, the SKAT-O approach (Lee et al., 2012), which is statistically efficient regardless of the direction and effect of the variants tested.

**Figure 1:**
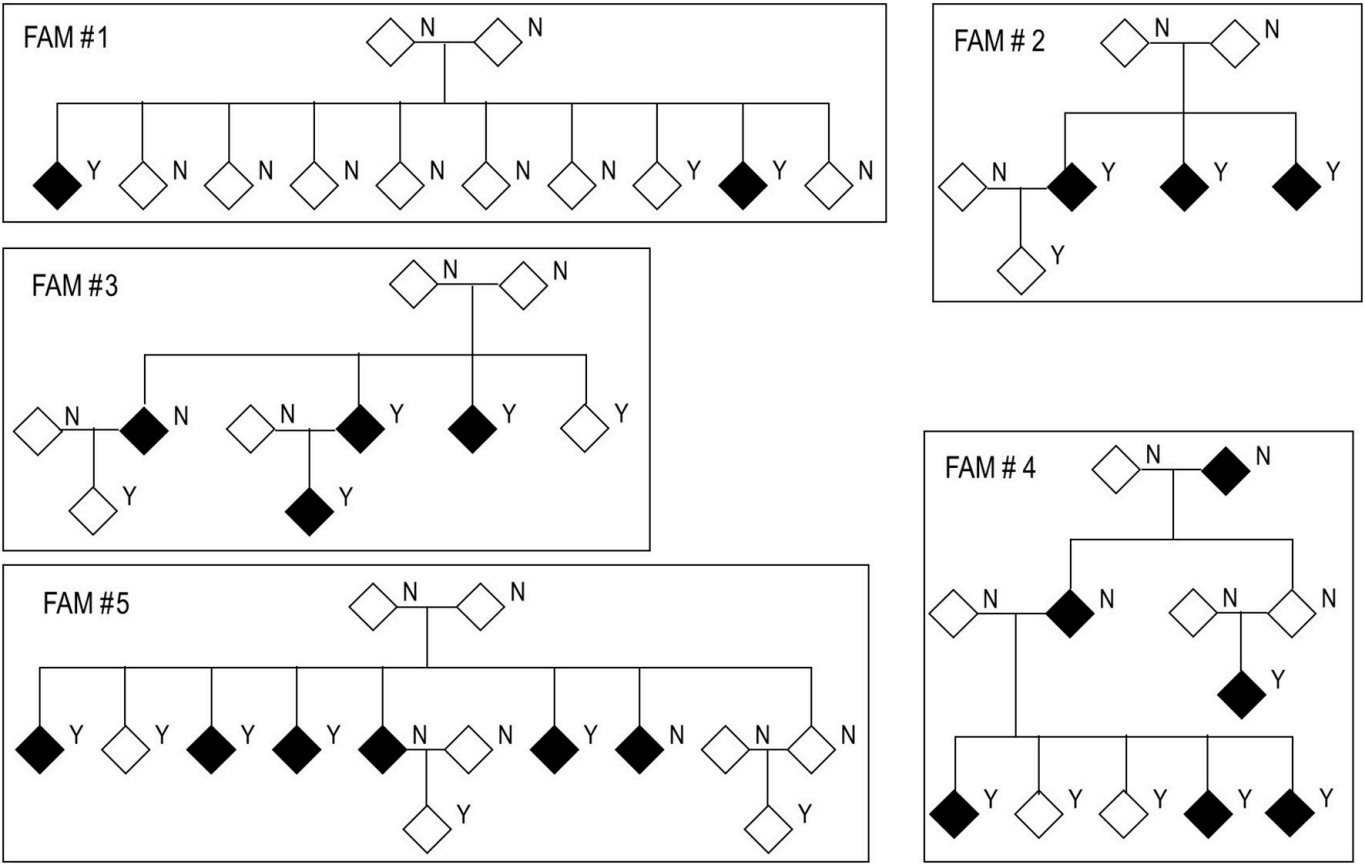
Structure of families used in this study. Black diamonds represent cases and white diamonds represent controls. Y: genetic data available. N: no genetic data available.

All these methods were initially designed for unrelated case-control studies; but considering the rarity of these variants, large datasets are required to achieve statistical power (Laird and Lange, 2006). Alternatively, family-based studies in which several family members share the same phenotype may provide more statistical power than regular case-controls studies (Li et al., 2006; Cirulli and Goldstein, 2010; Kazma and Bailey, 2011; Ott et al., 2011). Pioneering methods for gene-based analyses in familial datasets are based on the transmission disequilibrium test (TDT–Spielman et al., 1993) which uses the marker genotype of an affected child and genotypes of the parents to test for association (Laird et al., 2000; Horvath et al., 2001; Ott et al., 2011; De et al., 2013; Ionita-Laza et al., 2013). TDT works under the paradigm of Mendel's laws to determine which marker in the affected offspring is responsible for the phenotype (Zöllner et al., 2004). TDT methods have been extended to test rare-variants by grouping information across multiple variants within a genomic region (He et al., 2014). However, these methods were still not valid for incomplete or nuclear families that have several affected offspring. Considering the late-onset nature of Alzheimer disease it is often difficult to obtain genetic information from parents (to conform trios) or nuclear family units. The typical pedigree in familial LOAD represents incomplete, large familial units (Figure 1). Most of the early software for gene-based family-based studies were not suitable for complex pedigrees like those observed in Alzheimer studies. In recent years gene-based methods, whether referring to collapsing, variance-component, or transmission disequilibrium tests, have been adapted to account for complex family structure in its gene-based calculations. Among the software that can manage large pedigrees we find SKAT (Wu et al., 2011), FSKAT (Yan et al., 2015), GSKAT (Wang et al., 2013), RV-GDT (He et al., 2017), EPACTS (http://genome.sph.umich.edu/wiki/EPACTS), FarVAT (Choi et al., 2014), PedGene (Schaid et al., 2013), and RareIBD (Sul et al., 2016).

In this study, we wanted to evaluate the performance of the eight most common gene-based family-based methods available by using a real dataset, over 250 multiplex families affected with Alzheimer disease, under different conditions and models. We simulated multiple scenarios in which candidate variants in the same gene perfectly segregates with disease status to rank the different programs and models. We also tested the performance of these tests for identifying known causal genes for AD in our cohort. Finally, we performed genome-wide analyses to evaluate the power of each of these tests. Altogether, we discuss the pros and cons of each method that can be informative for other investigators performing similar analyses: complex diseases in complex, incomplete, large families. We want to emphasize that although this work focused on AD, the information extracted from this work can be applied to other complex traits as well. Finally, based on the results from the methods analyzed, we present some candidate genes for AD.

## Materials and methods

### Cohort

The LOAD families included in this study originated from two cohorts: Washington University School of Medicine (WUSM; *n* = 1,144) and Alzheimer Disease Sequencing Project (ADSP; *n* = 91) (Table 1).

**Table 1:** Demographic data for the familial dataset employed in this study.

	**N**	^*^Age ± SD	^*^Age range	**% Fe**	**% APOE4**
Cases	824	73 ±7	48–99	63	73
Controls	411	83 ± 9	39–104	59	51
Total	1235	77 ± 10	39–104	61	65

* *Age At Onset (AAO) for cases and Age at Last Assessment (ALA) for controls*.

#### WUSM cohort

Samples from the Washington University School of Medicine (WUSM) cohort were recruited by either the Charles F. and Joanne Knight Alzheimer's Disease Research Center (Knight ADRC) at the WUSM in Saint Louis or the National Institute on Aging Genetics Initiative for Late-Onset Alzheimer's Disease (NIA-LOAD). This study was approved by each recruiting center's Institutional Review Board and research was carried out in accordance with the approved protocol. Written informed consent was obtained from participants and their family members by the Clinical and Genetics Core of the Knight ADRC. The approval number for the Knight ADRC Genetics Core family studies is 201104178. The NIA-LOAD Family Study has recruited multiplex families with two or more siblings diagnosed with LOAD across the United States. A description of these samples has been reported previously (Wijsman et al., 2011; Cruchaga et al., 2012; Fernández et al., 2017). We selected individuals for sequencing from families in which APOEε4 did not segregate with disease status, and in which the proband of the family did not carry any known mutation in *APP, PSEN1, PSEN2, MAPT, GRN*, or *C9orf72* (described previously; Cruchaga et al., 2012).

#### ADSP cohort

The Alzheimer's Disease Sequencing Project (ADSP) is a collaborative work of five independent groups across the USA that aims to identify new genomic variants contributing to increased risk for LOAD (https://www.niagads.org/adsp/content/home). During the discovery phase, ADSP generated whole genome sequence (WGS) data from members of multiplex LOAD families, and whole exome sequence (WES) data from a large case-control cohort. These data are available to qualified researchers through the database of Genotypes and Phenotypes (https://www.ncbi.nlm.nih.gov/gap Study Accession: phs000572.v7.p4).

The familial cohort of the ADSP consists of 582 individuals from 111 multiplex AD families from European-American, Caribbean Hispanic, and Dutch ancestry (details about the samples are available at NIAGADS). We downloaded raw data (.sra format) from dbGAP for 143 IDs (113 cases and 23 controls) from 37 multiplex families of European-American ancestry that were incorporated with the WUSM cohort.

### Sequencing

Samples were sequenced using either whole-genome sequencing (WGS, 12%) or whole-exome sequencing (WES, 88%). Exome libraries were prepared using Agilent's SureSelect Human All Exon kits V3 and V5 or Roche VCRome (Table 2). Both WES and WGS samples were sequenced on a HiSeq2000 with paired end reads, with a mean depth of coverage of 50 × to 150 × for WES and 30 × for WGS. Alignment was conducted against GRCh37.p13 genome reference. Variant calling was performed separately for WES and WGS following GATK's 3.6 Best Practices (https://software.broadinstitute.org/gatk/best-practices/) and restricted to Agilent's V5 kit plus a 100 bp of padding added to each capture target end. We used BCFTOOLS (https://samtools.github.io/bcftools/bcftools.html) to decompose multiallelic variants into biallelic prior to variant quality control. Variant Quality Score Recalibration (VQSR) was performed separately for WES and WGS, and for SNPs and INDELs. Only those SNPs and indels that fell above the 99.9 confidence threshold, as indicated by WQSR, were considered for analysis; variants within low complexity regions were removed from both WES and WGS and variants with a depth (DP) larger than the average DP + 5 SD in the WGS dataset were removed. At this point SNPs and indels from WES and WGS datasets were merged into one file. Non-polymorphic variants and those outside the expected ratio of allele balance for heterozygosity calls (ABHet = 0.3–0.7) were removed. Additional hard filters implemented included quality depth (*QD* ≥ 7 for indels and *QD* ≥ 2 for SNPs), mapping quality (*MQ* ≥ 40), fisher strand balance (*FS* ≥ 200 for indels and *FS* ≥ 60 for SNPs), Strand Odds Ratio (SOR ≥ 10 for Indels and SOR ≥ 3 for SNPs), Inbreeding Coefficient (IC ≥ −0.8 for indels) and Rank Sum Test for relative positioning of reference vs. alternative alleles within reads (RPRS ≥ −20 for Indels and RPRS ≥ −8 for SNPs) (Figure S1). We used PLINK1.9 (https://www.cog-genomics.org/plink2/ibd) to remove variants that were out of Hardy Weinberg equilibrium (*p* < 1 × 10^−6^), with a genotype calling rate below 95%, with differential missingness between cases vs. controls, WES vs. WGS, or among different sequencing platforms (*p* < 1 × 10^−6^).

**Table 2:** Number of samples for which whole genome sequencing (WGS) or whole exome sequencing (WES) was performed, with detail of the exon library kits employed in this study.

**Exon library kit**	**WGS**	**WES**
WGS	153	
Agilent's SureSelect Human All Exon kits V3	0	28
Agilent's SureSelect Human All Exon kits V5	0	665
Roche VCRome	0	389
Total	153	1,082

Samples with more than 10% of missing variants (four samples) and whose genotype data indicated a sex discordant from the clinical database (three samples) were removed from the dataset. Individual and familial relatedness was confirmed using identity-by-descent (IBD) calculations, an existing GWAS dataset for these individuals, and the pedigree information. Because many of the ADSP families were also recruited from the NIA-LOAD repository there is a certain overlap (48 individuals) between the WUSM and the ADSP familial cohorts; we kept the duplicate that had better genotyping rate after QC. Principal Component Analysis (PCA) was calculated to corroborate ancestry and restrict our analysis to only samples from European American origin. Functional impact and population frequencies of variants were annotated with SnpEff (Cingolani et al., 2012). For this analysis, only SNVs with a minor allele frequency (MAF) below 1%, as registered in ExAC (Lek et al., 2016), were tested.

We excluded families carrying a known pathogenic mutation in any of the Mendelian genes for Alzheimer disease, Frontotemporal Dementia, or Parkinson disease (Fernández et al., 2017). We restricted the selection of families to those with at least one case and one control in the family, and we excluded any participants that were initially clinically diagnosed with AD but had a different diagnosis after pathological examination. Finally, our dataset consisted of 1,235 non-hispanic whites (NHW), 824 cases and 411 controls, from 285 different families (Table 1, Table S1). Of these 1,235 individuals, 1144 originated from WUSM and 91 were from ADSP.

### Study design and analysis

**Figure 2:**
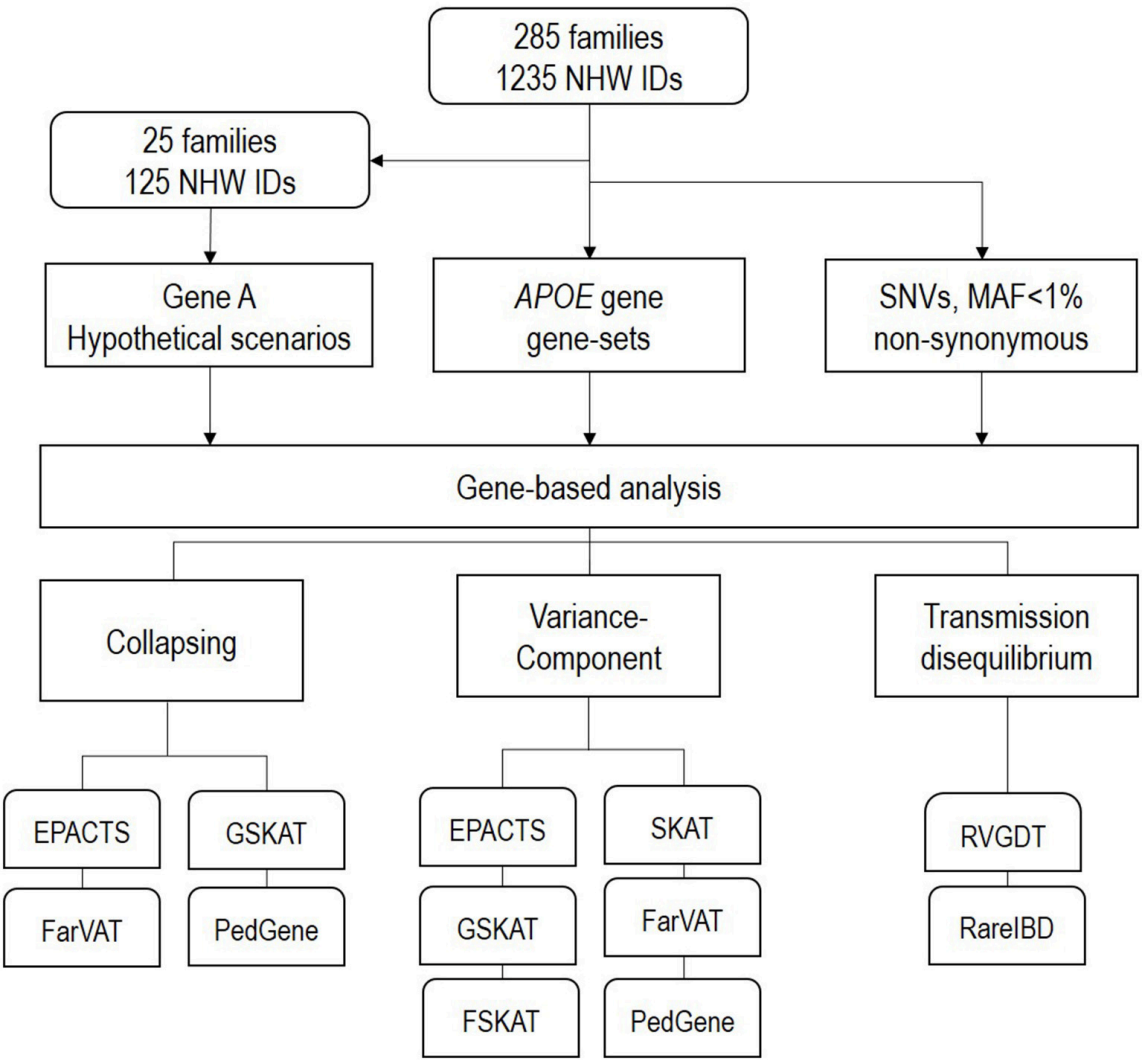
Schematic design of the analysis performed in this study.

The goal of this study was to test the performance and power of different gene-based family-based methods currently available, using a real dataset consisting of 1,235 non-hispanic white individuals from 285 families densely affected with AD. We created three different scenarios to test (Figure 2). First, using the real phenotype and pedigree structure from 25 of the 285 families, we generated a synthetic dataset with multiple variants and families with perfect segregation. Second, we evaluated different variant-combinations for the *APOE* gene. Third, we performed genome-wide gene-based analysis of only nonsynonymous SNPs with a MAF <1%. For each one of these scenarios we evaluated the performance of the different gene-based methods (collapsing, variance-component, and transmission disequilibrium) from the following family-based packages: SKAT (Wu et al., 2011), FSKAT (Yan et al., 2015), GSKAT (Wang et al., 2013), RVGDT (He et al., 2017), EPACTS (http://genome.sph.umich.edu/wiki/EPACTS), FarVAT (Choi et al., 2014), PedGene (Schaid et al., 2013), RareIBD (Sul et al., 2016). Some of these software offer the option to run different gene-based algorithms; e.g., GSKAT, EPACTS, FarVAT or PedGene can run collapsing and variance-component tests; therefore, we ran a total of 25 models (Table 3). The details of each one of these scenarios are described next.

**Table 3:** Relationship of programs and models tested according to their main features and kinship matrix that they use.

	**Collapsing**			**Variance-component**		**Combined**	**Transmission-disequilibrium**	**Kinship**		
	**Burden**	**CMC**	**VT**	**C-ALPHA**	**SKAT**	**SKATO**		**BN**	**IBS**	**Ped**
EPACTS		X	X		X			X		
RVGDT							X			
SKAT-v2					X			X	X	X
GSKAT	X				X					X
FSKAT					X					X
FarVat-Adj	X	X		X		X				
FarVat-BLUP	X	X		X		X				
Pedgne	X				X					
RareIbd							X			

#### Simulated data

We selected 25 representative families from our entire dataset for which there were genotypic data for three to seven members (Table S2). We used the existing family structure and phenotypes of these families, and a simulated gene called “GENE-A” containing five variants. We generated several scenarios in which different numbers of families presented perfect segregation with disease status for a variant in GENE-A (Table 4, Table S2). First, we considered a scenario in which only the first five families of the dataset were included in the analyses and each family presented a different perfectly segregating variant of GENE-A [scenario 5 family carriers (FC) and 0 non-carriers (FNC): 5FC × 0FNC]. Second, we generated additional scenarios in which we kept the same five families as carriers of segregating variants in GENE-A, and added five (scenario 5FC × 5FNC), ten (scenario 5FC × 10FNC), 15 (scenario 5FC × 15FNC), and 20 (scenario 5FC × 20FNC) families that were not carriers of any variant in GENE-A. Then, we considered four scenarios of 25 families in which each new scenario added families who were carriers of a segregating variant in GENE-A. We started with the scenario 5FC × 20FNC, then we simulated 10 families who had carriers and 15 families who were non-carriers (scenario 10FC × 15FNC), 15 families with carriers and 10 families who were non-carriers (scenario 15FC × 10FNC), 20 families with carriers and five families who were non-carriers (scenario 20FC × 5FNC) and concluded with a scenario in which all 25 families were carriers of one out of the possible five segregating variants in GENE-A (scenario 25FC × 0FNC). We tested each of these scenarios with all previously mentioned gene-based methods and software to evaluate their power to associate perfect segregating variants with disease.

**Table 4:** Representation of the segregation pattern of the simulated gene.

	**GENE-A**				
	**SNP1**	**SNP2**	**SNP3**	**SNP4**	**SNP5**
Fam1	**1**	0	0	0	0
Fam2	0	**1**	0	0	0
Fam3	0	0	**1**	0	0
Fam4	0	0	0	**1**	0
Fam5	0	0	0	0	**1**
Fam6	**1**	0	0	0	0
Fam7	0	**1**	0	0	0
Fam8	0	0	**1**	0	0
Fam9	0	0	0	**1**	0
Fam10	0	0	0	0	**1**
Fam11	**1**	0	0	0	0
Fam12	0	**1**	0	0	0
Fam13	0	0	**1**	0	0
Fam14	0	0	0	**1**	0
Fam15	0	0	0	0	**1**
Fam16	**1**	0	0	0	0
Fam17	0	**1**	0	0	0
Fam18	0	0	**1**	0	0
Fam19	0	0	0	**1**	0
Fam20	0	0	0	0	**1**
Fam21	**1**	0	0	0	0
Fam22	0	**1**	0	0	0
Fam23	0	0	**1**	0	0
Fam24	0	0	0	**1**	0
Fam25	0	0	0	0	**1**

*One (1) means that all cases within the family are carriers of the variant. Zero (0) means that the variant is not present in that family*.

#### Candidate genes

*APOE* is the largest genetic risk factor for Alzheimer's disease. The allelic combination of two SNPs, rs429358 (APOE 4; 19:45411941:T:C), and rs7412 (APOE 2: 19:45412079:C:T), determines one of the three major isoforms of APOE protein, ε2, ε3, or ε4. The dosage of these isoforms determines a person's risk for AD, from having a protective effect in the cases of APOE ε2/ε2 (OR 0.6) or ε2/ε3 (OR 0.6) to different degrees of increased risk according to the number of copies of the ε4 allele (ε2/ε4, OR 2.6; ε3/ε4, OR 3.2; ε4/ε4, OR 14.9) (Farrer et al., 1997). We tested the power of all previously mentioned gene-based methods and software to detect the association of *APOE* gene with disease in our entire dataset (*N* = 1,235) under different conditions. We first tested all polymorphic variants (nonsynonymous with MAF <1%) in the *APOE* gene, next we tested only those variants considered to have a high or moderate effect on the protein including rs429358 and rs7412, then we tested high and moderate effect variants alone, and finally tested rs429358 and rs7412 alone.

#### Genome-wide analyses

We performed gene-based burden analyses on a genome-wide level in our entire dataset (families *n* = 285; samples *N* = 1,235) to evaluate the power of each of the previously described methods to detect novel genes significantly associated with disease; only single nucleotide variants (SNVs) with a minor allele frequency equal to or below 1% (MAF ≤ 1%), based on the EXAC dataset (Lek et al., 2016), and with a predicted high or moderate effect, according to SnpEff (Cingolani et al., 2012), were included in the analysis. Quantile-Quantile (QQ) plots from gene-based *p*-values were generated with the R package “ggplot2” (Wickham, 2009). We also evaluated the correlations between these methods using Pearson correlation (Pc) and Spearman correlation (Sc) tests of the log of the *p*-values using R v3.4.0 (R Core Team, 2017). Pc evaluates the linear relationship between two continuous variables whereas Sc evaluates the monotonic relationship between two continuous or ordinal variables.

### Software tested

An accompanying supporting file (Supplementary Material) provides a summary of the code employed to run each of the programs described below.

#### GSKAT

GSKAT (Wang et al., 2013) is among the first R packages developed with the goal of extending burden and kernel-based gene set association tests for population data to related samples with binary phenotypes. To handle the correlated or clustered structure in the family data, GSKAT fits a marginal model with generalized estimated equations (GEE). The basic idea of GEE is to replace the covariance matrix in a generalized linear mix model (GLMM) with a working covariance matrix that reflects the cluster dependencies. Accordingly, GSKAT blends the strengths of kernel machine methods and generalized estimating equations (GEE) to test for the associations between a phenotype and multiple variants in a SNP set. We ran GSKAT correcting for sex and first two PCs.

#### SKAT

The sequence kernel association test SKAT (Wu et al., 2011) is an R package initially designed for case-control analyses. Later they incorporated the Efficient Mixed-Model Association eXpedited (EMMAX) algorithm (Kang et al., 2010; Zhou and Stephens, 2012) which allows for performing family-based analyses. EMMAX simultaneously corrects for both population stratification and relatedness in an association study by using a linear mixed model with an empirically estimated relatedness matrix to model the correlation between phenotypes of sample subjects. The efficient application of the EMMAX algorithm depends on appropriate estimates of the variance parameters. Relatedness matrices can be calculated based on pedigree structure or estimated from genotype data. For the latter different methods have been proposed. Relatedness can be estimated using those alleles that have descended from a single ancestral allele, i.e., those that are Identical by Descent (IBD), or using the Balding-Nichols (BN) method (Balding and Nichols, 1995) which explicitly models current day populations via their divergence from an ancestral population specified by Wright's *F*_*st*_ statistic. We ran SKAT v1.2.1, in R v3.3.3, using the option SKAT_Null_EMMAX correcting for sex and first two PCs and we tested four different kinship matrices: pedigree, IBS, BN and a BN-based kinship matrix (HR) that the EPACTS software constructs (Table S3).

#### FSKAT

FSKAT (Yan et al., 2015), also an R package, is based on a kernel machine regression and can be considered an extension of the sequence kernel association tests (SKAT and famSKAT) for application to family data with dichotomous traits. FSKAT is based on a GLMM framework. Moreover, because it uses all family samples, FSKAT claims to be more powerful than SKAT which uses only unrelated individuals (founders) in the family data. FSKAT constructs a kinship matrix based on pedigree relationships using the R kinship library. We ran FSKAT correcting for sex and first two PCs.

#### EPACTS

Efficient and Parallelizable Association Container Toolbox (EPACTS) is a stand-alone software that integrates several gene-based statistical tests (CMC, VT, and SKAT) and adapts them to work with complex families by using EMMAX (https://genome.sph.umich.edu/wiki/EPACTS). EPACTS generates a kinship matrix based on the BN algorithm and also annotates the genotypic input file and offers filtering tools (frequency and predicted effect of variants) for easier user-selection of variants that go into gene-based analyses. Nonetheless, we used the same set of variants as in the other tests to run our analysis with EPACTS, correcting for sex and first two PCs.

#### FarVAT

The Family-based Rare Variant Association Test (FarVAT) (Choi et al., 2014) provides a burden and a variance component test (VT) for extended families and extends these approaches to the SKAT-O statistic. FarVAT assumes that families are ascertained based on the disease status if family members and compares minor allele frequencies between affected and unaffected individuals. FarVAT is implemented with C++ and is computationally efficient. Additionally, if genotype frequencies of affected and unaffected samples are compared to detect genetic associations, it has been shown that the statistical efficiency can be improved by modifying the phenotype; and so FarVAT uses prevalence (Lange and Laird, 2002) or Best Linear Unbalanced Predictor (BLUP) (Thornton and McPeek, 2007) as covariate to modify the genotype.

#### PedGene

PedGene (Schaid et al., 2013) is an R package that extends burden and kernel statistics to analyze binary traits in family data using large-scale genomic data to calculate pedigree relationships. To derive the kernel association statistic and the burden statistic for data that includes related subjects, they take a retrospective view of sampling with the genotypes considered random.

#### RVGDT

The Rare Variant Generalized Disequilibrium Test (RVGDT) (He et al., 2017), implemented with Python, differs from the previous methods presented. Instead of using a kernel method to evaluate variants, it uses the generalized disequilibrium test (GDT) which tests genotype differences in all discordant relative pairs to assess associations within a family (Chen et al., 2009). The rare-variant extension of GDT (RVGDT) aggregates a single-variant GDT statistic over a genomic region of interest, which is usually a gene (He et al., 2017). We ran RVGDT correcting for sex and first two PCs.

#### RareIBD

The developers claim RareIBD (Sul et al., 2016) to be a program without restrictions on family size, type of trait, whether founders are genotyped, or whether unaffected individuals are genotyped. The method is inspired by non-parametric linkage analysis and looks for rare variants with segregation patterns among affected and unaffected individuals that are different from the predicted distributions based on Mendelian inheritance and computes a statistic measuring the difference.

## Results

### Simulated dataset

Results from the simulated dataset indicate that RVGDT, rareIBD, and collapsing-based methods (Burden, CMC, and CLP) provided more statistical power than the variance-component methods to detect associations of perfectly segregating variants with disease status (Table 5).

**Table 5:** Gene-based *p*-values for the simulated dataset under different scenarios for the gene-based methods tested in the subset of 25 families.

**SET**	**GSKAT**	**FSKAT**	**SKAT**	**RVGDT**	**PedGene**		**Rare IBD**	EPACTS^*^	**FarVAT**					**FarVAT-BLUP**				
					**SKAT**	**Burden**		**SKAT**	**CMC**	**CLP**	**CALPHA**	**Burden**	**SKATO**	**CMC**	**CLP**	**CALPHA**	**Burden**	**SKATO**
5FC × 0FNC	0.236	NA	0.141	0.004	0.301	0.003	<1 × 10^−5^	NA	5.42 × 10^−6^	4.66 × 10^−6^	NA	NA	NA	3.93 × 10^−9^	3.06 × 10^−9^	NA	NA	NA
5FC × 5FNC	0.235	0.124	0.023	0.002	0.123	7.99 × 10^−4^	<1 × 10^−5^	NA	0.004	0.005	NA	NA	NA	2.10 × 10^−5^	4.00 × 10^−5^	NA	NA	NA
5FC × 10FNC	0.354	0.338	0.112	0.005	0.079	7.99 × 10^−4^	<1 × 10^−5^	NA	0.032	0.036	NA	NA	NA	7.71 × 10^−4^	1.01 × 10^−3^	NA	NA	NA
5FC × 15FNC	0.377	0.359	0.202	0.005	0.095	0.002	<1 × 10^−5^	NA	0.062	0.061	NA	NA	NA	0.002	2.84 × 10^−3^	NA	NA	NA
5FC × 20FNC	0.377	0	0.201	0.006	0.114	0.003	<1 × 10^−5^	0.321	0.073	0.075	0.670	0.075	0.134	0.002	2.40 × 10^−3^	0.132	0.002	0.005
10FCA × 15FNC	0.083	0	0.028	9 × 10^−4^	0.004	2.65 × 10^−6^	<1 × 10^−5^	0.047	0.005	0.008	0.272	0.008	0.017	6.81 × 10^−6^	1.33 × 10^−5^	0.013	1.33 × 10^−5^	3.62 × 10^−5^
15FC × 10FNC	0.014	0	0.005	9 × 10^−4^	0.001	1.77 × 10^−9^	<1 × 10^−5^	0.051	1.72 × 10^−6^	6.31 × 10^−5^	0.024	6.31 × 10^−5^	1.30 × 10^−4^	4.26 × 10^−11^	3.27 × 10^−9^	0.001	3.27 × 10^−9^	8.93 × 10^−9^
20FC × 5FNC	0.002	0	0.002	9 × 10^−4^	0.002	1.30 × 10^−9^	<1 × 10^−5^	0.039	1.48 × 10^−11^	7.85 × 10^−7^	0.024	7.85 × 10^−7^	1.14 × 10^−6^	6.12 × 10^−18^	2.12 × 10^−12^	6.32 × 10^−4^	2.12 × 10^−12^	2.54 × 10^−10^
25FC × 0FNC	3 × 10^−4^	0	0.001	9 × 10^−4^	0.001	1.42 × 10^−10^	<1 × 10^−5^	0.033	1.55 × 10^−19^	4.44 × 10^−8^	0.025	4.44 × 10^−8^	7.06 × 10^−8^	4.59 × 10^−29^	4.58 × 10^−15^	5.10 × 10^−4^	4.58 × 10^−15^	2.54 × 10^−10^

*Simulated scenarios: 5FC, five families carrier of variants within the hypothetical gene; 5FC × 5FNC, five families carrier of variants within the hypothetical gene and five families non-carrier of variants within the hypothetical gene; 5FC × 10FNC, five families carrier of variants within the hypothetical gene and 10 families non-carrier of variants within the hypothetical gene; 5FC × 15FNC, five families carrier of variants within the hypothetical gene and 15 families non-carrier of variants within the hypothetical gene; 5FC × 20FNC, five families carrier of variants within the hypothetical gene and 20 families non-carrier of variants within the hypothetical gene; 10FC × 15FNC, 10 families carrier of variants within the hypothetical gene and 15 families non-carrier of variants within the hypothetical gene; 15FC × 10FNC, 15 families carrier of variants within the hypothetical gene and 10 families non-carrier of variants within the hypothetical gene; 20FC × 5FNC, 20 families carrier of variants within the hypothetical gene and five families non-carrier of variants within the hypothetical gene; 25FC, 25 families carrier of variants within the hypothetical gene*.

* *we tested SKAT, CMC, and VT on EPACTS, but CMC and VT reported all NA values so data is not shown*.

In a hypothetical scenario of five families in which each family presented perfect segregation with disease status for a different variant within the same gene (5FC × 0NFC), transmission-disequilibrium based methods evaluated this association as significant (even after multiple test correction; e.g., RVGDT *p* = 0.004; *p*-value after multiple test correction 0.004 × 9 = 0.036). RVGDT reached a ceiling *p*-value of 1 × 10^−4^; at 10 families with carriers (FC) plus 15 families of non-carriers (FNC). RVGDT was unable to produce a *p* < 9 × 10^−4^, therefore it is not possible to rank or determine the significance of genes that reach this limit. Similarly, RareIBD reports the same *p*-value for all simulated scenarios, which may be an artifact or a flaw of the program. Collapsing-based methods (Burden, CMC and CLP) started with significant *p*-values for the 5FC × 0NFC scenario, but as we added FNC in the analyses, the associations became less significant. Then as we increased the number of FC of segregating variants, the associations became more significant. In our analyses, most of the variance-component tests could not work with the scenarios containing only five families carrying the segregating variant; most of the tests only provided *p*-values once 25 families were included in the analyses (5FC × 20FNC). After that, as we increased the number of FC of segregating variants, the *p*-values became smaller. SKAT required 15FC × 10FNC to report nominally significant *p*-values, GSKAT required 20FC × 5FNC to report statistically significant *p*-values, FarVAT-CALPHA did not generate significant *p*-values unless we used the BLUP correction; FarVAT SKATO reported *p*-values that were significant at 15FC × 10FNC, and at 5FC × 20FNC if we used the BLUP correction. *P*-values from EPACTS-SKAT were not statistically significant after multiple test correction. FSKAT did not deal well with perfectly segregating scenarios; it did not provide *p*-values for a scenario of only five families all carriers of the segregating variant (5FC × 0FNC–FSKAT *p*-value = NA), and after five families carrying a segregating variant, the program saturated giving no *p*-value.

Overall, Transmission-disequilibrium tests and collapsing tests were the models that identified the simulated segregating variants as associated with the phenotype; the CMC model provided by FarVAT-BLUP was the one providing most genome-wide significant *p*-values, even in the 5FC × 0FNC scenario.

### Candidate genes-APOE

We examined the performance of four gene-sets generated for the *APOE* gene with the 22 family-based gene-based methods in our entire familial cohort. Neither the entire set of polymorphic variants (set “gene” in Table 6) nor the set including only rare nonsynonymous variants (set “HM” in Table 6) confer risk for these families. The association seems to be driven by the common *APOE* ε2 and ε4 variants, since only when these were included, either alone (set “ε2ε4” in Table 6) or in conjunction with the rest of the rare nonsynonymous variants (set “HM-ε2ε4” in Table 6) did most of the tests yield a significant *p*-value (after multiple test correction). Only EPACTS-SKAT did not report the *APOE* ε2 and ε4 variants as significantly associated, after multiple test correction, within our dataset (Table 6). The most significant association for *APOE* ε2 and ε4 variants was reported by FarVAT-CMC test.

**Table 6:** Gene-based *p*-values for the *APOE* gene under different gene-set scenarios for the gene-based methods tested in the entire dataset (*N* = 1235, 285 families).

***APOE***	**N**	**GSKAT**	**FSKAT**	**SKAT**	**RVGDT**	**PedGene**		**Rare IBD**	EPACTS^*^	**FarVAT**					**FarVAT-BLUP**				
						**SKAT**	**Burden**		**SKAT**	**CMC**	**CLP**	**CALPHA**	**Burden**	**SKATO**	**CMC**	**CLP**	**CALPHA**	**Burden**	**SKATO**
gene	19	0.035	0.037	0.061	0.164	**0.008**	0.515	0.712	0.205	0.053	0.379	**0.003**	0.379	**0.005**	0.036	0.311	0.017	0.311	0.034
HM-ε2ε4	4	**0.003**	**0.002**	**0.001**	**0.005**	0.412	0.414	0.359	0.020	**7.87** × **10**^−15^	0.420	**4.99** × **10**^−4^	0.420	**0.001**	**3.73** × **10**^−14^	0.275	**3.99** × **10**^−4^	0.275	**6.99** × **10**^−4^
HM	2	0.067	0.089	0.048	0.237	0.177	0.177	0.741	0.022	0.028	0.052	0.014	0.052	0.018	0.053	0.090	0.024	0.090	0.031
ε2ε4	2	**0.005**	**0.002**	**0.003**	**0.004**	0.849	0.855	**0.002**	0.024	**7.87** × **10**^−15^	**0.002**	**0.002**	**0.002**	**0.003**	**3.73** × **10**^−14^	**0.002**	**0.001**	**0.001**	**0.001**

*In the analysis, only nonsynonymous variants (only SNVs) with a MAF < 0.01, and the APOE ε2 and ε4, were considered and we adjusted by sex and PCAs. Highlighted in bold, significant p-values after multiple test correction*.

*gene, set of 19 polymorphic variants within APOE gene, including APOE ε2 and ε4 variants; HM-ε2ε4, set of variants considered HIGH or MODERATE including APOE ε2 and ε4 variants; HM, set of variants considered HIGH or MODERATE without APOE ε2 and ε4 variants; ε2ε4, APOE ε2 and ε4 variants alone. N, number of variants that went into analysis*.

* *We tested SKAT, CMC, and VT on EPACTS, but CMC and VT reported all NA values so data is not shown*.

### Genome-wide analyses

**Figure 3:**
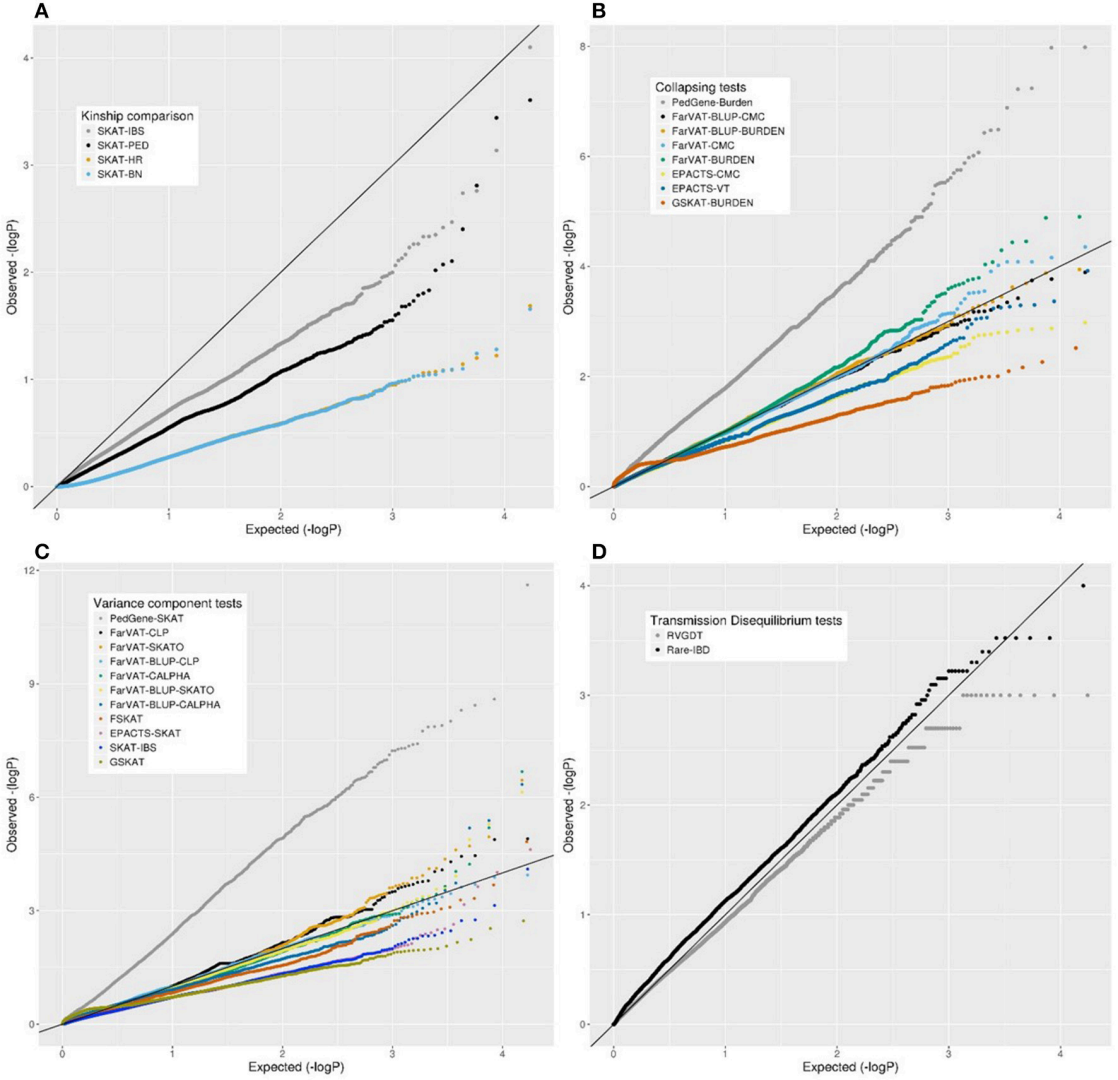
Quantile-quantile (QQ) plots from different family-based gene-based methods for all nonsynonymous variants with a MAF <1% in our family-based dataset. **(A)** Comparison of SKAT test using different kinship matrices: pedigree calculation (PED), Identity By Similarity (IBS) estimation, Balding-Nichols (BN) estimation, and the kinship generated by EPACTS (HR). **(C)** Comparison of different collapsing tests: GSKAT, EPACTS, FarVAT, and PedGene. **(B)** Comparison of different variance-component gene-based methods: GSKAT, FSKAT, SKAT, EPACTS, FarVAT, and PedGene. **(D)** Comparison of transmission disequilibrium tests: RVGDT and RareIBD.

Overall, we examined eight software and over 22 algorithms for genome-wide association analyses in our extended family dataset of 285 families and 1,235 non-hispanic white individuals. We only included in the analyses nonsynonymous SNPs with a MAF ≤ 1% and we corrected for sex and first two PCs. All 22 algorithms were run using the same input data. The results for these 22 algorithms are described, grouped per category, in the following sections. First, we compare the correction effect provided by four kinship matrices (Figure 3A). Second, we compare the performance of nine variance-component software and algorithms (Figure 3B). Third is the comparison of eight collapsing software and algorithms. Fourth, we compare two transmission-disequilibrium tests. We conclude the results section by providing a summary of the pros and cons encountered while running these methods. Overall, most of the results from the gene-based methods tested seemed quite deflated. Only PedGene, FarVAT and Rare-IBD seemed to provide values closer to or above the expected under the null hypothesis. The most efficient in terms of power and *p*-value inflation appears to be FarVAT with BLUP correction.

#### Kinship matrices

We tested the correction provided by four kinship matrices using the SKAT method with EMMAX correction implemented in the R package SKATv2. The four kinship matrices tested were pedigree calculation (PED), Identity By State (IBS) estimation, Balding-Nichols (BN) estimation, and the kinship generated by EPACTS (HR) which is also based on the BN algorithm (Figure 3A). Table S3 offers a comparison of these kinships for FAM#1 and FAM#2 of our simulated dataset. For these analyses, we ran the SKAT-EMMAX method in our entire dataset, gene-wide, and calculated a QQ plot and inflation factor (λ) to obtain a general ideal of the behavior of each matrix. Matrices based on the BN algorithm seemed to have a similar performance (SKAT-BN λ = 0.038, SKAT-HR λ = 0.039, Table 7) though their concordance was lower than expected considering they are based on the same algorithm [Pearson correlation (Pc) = 0.85; Spearman correlation (Sc) = 1]. Although the PED matrix generates a more restrictive correction than the IBS matrix (SKAT-PED λ = 0.36, SKAT-IBS λ = 0.67, Table 7), these two tests have a similar overall performance as the *p*-values for the different genes were highly correlated (*Pc* = 0.97; *Sc* = 0.98), making the PED matrix a good surrogate for the IBS matrix. Finally, there were clear performance differences between the BN-type matrices (BN and HR) and the IBS-type matrices (IBS and PED), exemplified by the different top candidate genes (*NR1D1* for BN-type matrices and *CHRD* for IBS-type matrices) and by the correlation algorithms (SAKT-IBS vs. SKAT-BN *Pc* = 0.8; *Sc* = 0.89). Overall, we found that the IBS matrix provided the best balance between covariance-correction and overcorrection in our dataset.

**Table 7:** Top results for all gene-based methods tested.

**Software**	**TEST**	**Top gene**	**Top *p*-value**	**Lambda**
PedGene	SKAT	*KANSL1L*	2.42 × 10^−12^	3.533
PedGene	Burden	*TTN*	1.04 × 10^−8^	2.997
GSKAT	Burden	*PCSK6*	3.04 × 10^−3^	1.704
GSKAT	SKAT	*NR1D1*	1.90 × 10^−3^	1.681
Rare-IBD	TDT	*SNTB2*	1.00 × 10^−4^	1.450
FarVAT-BLUP	CALPHA	*CHRD*	4.60 × 10^−07^	1.259
FarVAT	CALPHA	*CHRD*	2.09 × 10^−07^	1.152
FarVAT-BLUP	CLP	*NLRP9*	1.14 × 10^−4^	1.112
FarVAT-BLUP	SKATO	*CHRD*	7.37 × 10^−7^	1.101
FarVAT-BLUP	CMC	*IGHV1-69*	1.28 × 10^−4^	1.066
FarVAT-BLUP	Burden	*NLRP9*	1.14 × 10^−4^	1.031
FarVAT	SKATO	*CHRD*	3.54 × 10^−7^	1.016
FarVAT	CLP	*MAS1L*	1.25 × 10^−5^	1.000
RVGDT	TDT	*RTN3*	9.99 × 10^−4^	0.995
FarVAT	CMC	*HSD3B1*	4.40 × 10^−5^	0.993
FarVAT	Burden	*MAS1L*	1.25 × 10^−5^	0.985
EPACTS	VT	*PPAN-P2RY11*	1.20 × 10^−4^	0.954
FSKAT	SKAT	*CHRD*	2.00 × 10^−5^	0.938
EPACTS	CMC	*BTN2A2*	1.05 × 10^−3^	0.849
SKAT	IBS	*CHRD*	7.94 × 10^−5^	0.668
EPACTS	SKAT	*CHRD*	2.42 × 10^−5^	0.635
SKAT	PED	*CHRD*	2.47 × 10^−4^	0.360
SKAT	HR	*NR1D1*	2.06 × 10^−2^	0.039
SKAT	BN	*NR1D1*	2.21 × 10^−2^	0.038

*Top gene, p-value and lambda for each test is given, ordered by lambda value*.

#### Collapsing tests

The collapsing methods tested from four different software (PedGene, FarVAT, EPACTS and GSKAT) were Burden, CMC, and VT (Figure 3C). To compare the different tests we followed a similar approach as above, ran the different software with the same imputed file, and compared the λ.

In our analyses, the burden test by GSKAT presented the most deflated values; though the lambda does not illustrate this (GSKAT-Burden λ = 1.71, Table 7) because of the initial inflation among the low or non-significant genes. EPACTS-CMC (λ = 0.85) and EPACTS-VT (λ = 0.95) provided values closer to the expected, and although their QQ-plots appear to follow a similar trend, their correlation is low (*Pc* = 0.54; *Sc* = 0.68) and they reported different top genes. The Burden and CMC methods by FarVAT and FarVAT-BLUP provided *p*-values closest to the expected (FarVAT-Burden λ = 0.98; FarVAT-CMC λ = 0.99, FarVAT-BLUP-Burden λ = 1.03; FarVAT-BLUP-CMC λ = 1.07). The correlation for the gene *p*-values was higher between results generated by the same method (FarVAT-BLUP-CMC vs. FarVAT-BLUP-Burden *Pc* = 0.99; *Sc* = 0.96; FarVAT-CMC vs. FarVAT-Burden *Pc* = 0.98; *Sc* = 0.97) than between results generated using the same algorithm (FarVAT-BLUP-CMC vs. FarVAT-CMC *Pc* = 0.88; *Sc* = 0.8; FarVAT-BLUP-Burden vs. FarVAT-Burden *Pc* = 0.85; *Sc* = 0.77). PedGene in the burden model was the software that provided the most significant *p*-values; however, these were clearly inflated compared to the predicted *p*-values (Pedgene-Burden λ = 2.99, Table 7) and the results were not correlated with any other Collapsing test (Pc and Sc values < 0.1).

#### Variance component tests

**Figure 4:**
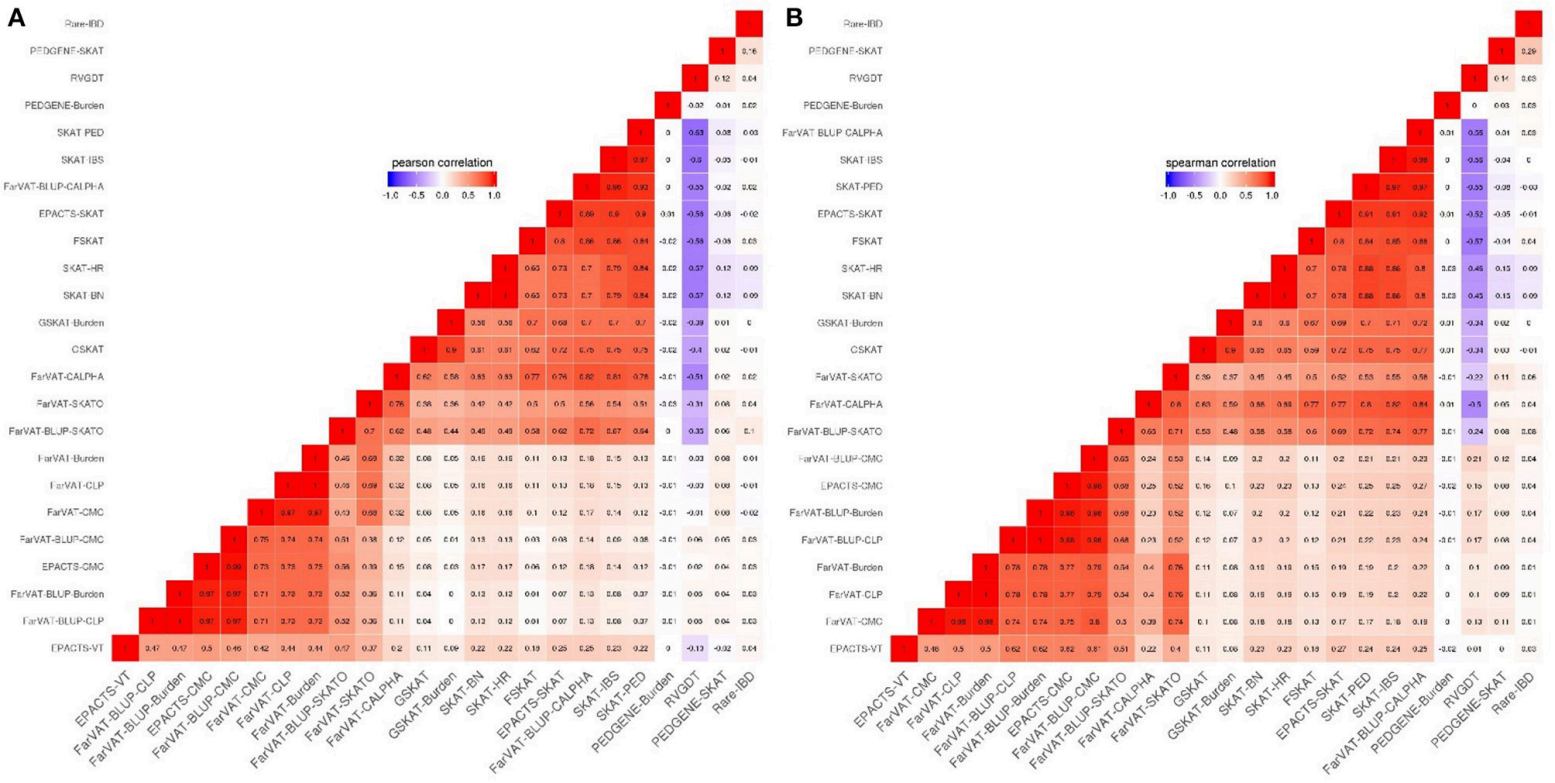
Correlation plots from different family-based gene-based methods for genes with a *p* ≤ 0.005. **(A)** Pearson correlation correlates genes according to their *p*-values. **(B)** Spearman correlation correlates genes according to their rankings.

This subset included all the Variance component-based methods available, CLP, CALPHA and SKAT, from six different software: PedGene, FarVAT, FSKAT, EPACTS, SKAT, and GSKAT (Figure 3C). GSKAT was the software that reported more deflated values, though the lambda does not illustrate this (GSKAT-SKAT λ = 1.681, Table 7) because of the initial inflation among the low or non-significant genes. GSKAT was followed by SKAT and EPACTS which showed similar λ and performance-values for each gene (*Pc* = 0.8, *Sc* = 0.8, Figure 4). The CLP, CALPHA, and SKATO methods by FarVAT and FarVAT-BLUP provided *p*-values closest to the expected (FarVAT-CLP λ = 1.00; FarVAT-CALPHA λ = 1.15; FarVAT-SKATO λ = 1.02, FarVAT-BLUP-CLP λ = 1.11; FarVAT-BLUP-CALPHA λ = 1.26; FarVAT-BLUP-SKATO λ = 1.10). FarVAT-CALPHA, FarVAT-SKATO, FarVAT-BLUP-CALPHA and FarVAT-BLUP-SKATO reported the same top candidate gene (*CHRD*) (Table 7), though the overall *p*-value correlation was lower than expected considering they are based on the same algorithm (FarVAT-SKATO vs. FarVAT-BLUP-SKATO *Pc* = 0.6, *Sc* = 0.7; FarVAT-CALPHA vs. FarVAT-BLUP-CALPHA *Pc* = 0.82 *Sc* = 0.82, Figure 4). On the other hand, despite the fact that FarVAT-CLP and FarVAT-BLUP-CLP had higher correlation (*Pc* = 0.85, *Sc* = 0.77), these two tests reported different top genes (FarVAT-CLP top gene is *MAS1L*, and FarVAT-BLIP-CLP top gene is *NLRP9*). PedGene in the SKAT model was the software that provided the most significant *p*-values, but these were clearly inflated (Pedgene-SKAT λ = 3.53, Table 7) and its correlation with other variance component tests was low to null (Pc and Sc values < 0.2).

#### Transmission disequilibrium tests

We tested two transmission disequilibrium tests, RVGDT and Rare-IBD, which were designed to account for large extended families of arbitrary structure (Figure 3D). Of these two, RVGDT was the test that more closely approached the expected under the null (λ = 0.99), whereas Rare-IBD provided slightly inflated *p*-values (λ = 1.450, Table 7). The correlation between these two methods was very low (Pearson correlation = 0.23, Spearman correlation = 0.17). A common issue with both methods was that we observed some stratification toward more significant *p*-values which made it difficult to determine a top significant gene.

#### Pros and cons of the different gene-based methods

Among all the methods tested, EPACTS and FarVAT are the most user-friendly, time-efficient and versatile software. EPACTS is an all-in-one package that annotates the input file, generates the kinship matrix and performs gene-based analysis under different conditions (minor allele frequency and predicted functionality of the variant) with only tag specification. In addition, the program can be run on a genome-wide basis or at a smaller scale given genes or regions specified by the user. FarVAT can generate the kinship matrix by either using the pedigree relationships or using the genetic relationship among individuals. It does not annotate the input file and requires that the user provide their own set of genes and variants per gene to analyze; it allows the user to choose between BLUP or prevalence to estimate and incorporate random effects on the phenotype. FarVAT has initial conditioning that only takes founder-based MAF, so when a genetic variant only has minor alleles in non-founders (offspring) these numbers will not be counted. This is a big limitation with respect to the other programs that take into account all variants regardless of their presence in founders or not. Since we only had genetic data for siblings for many of our families, so no genetic data for founders, we ran FarVAT with the “–freq all” option so that all variants would be included regardless if they were present in founders or not.

FSKAT, GSKAT, and SKAT require some R knowledge from the user, and are less flexible. For FSKAT and GSKAT the user has to provide a genotype, a phenotype, and a gene-set file. For SKAT the user has to additionally provide the kinship matrix. Because these programs were designed to run on a per gene basis, these take longer computational time to be run on a genome-wide level than EPACTS or FarVAT, even if the user parallelizes computation. PedGene is also an R package that requires a genotype, a phenotype file with complete pedigree information (to generate the kinship matrix), and a gene-set file. PedGene provides phenotype adjustment by logistic regression on the trait of interest, but it does not allow for extra covariates, which prohibits correction by multiple PCs or other variables. RVGDT is a Python based program, quite user-friendly since it is operated with simple command-line but is limited in its options. Similar to FSKAT, GSKAT, and SKAT, it is designed to be run on a per-gene basis for which loops and parallelization have to be set up for genome-wide testing. The same applies to RareIBD which requires a genotype, a phenotype, and a Kinship coefficient file for each gene that the user wants to test. For each gene the program first computes statistics for each founder within each family and then calculates the gene-based *p*-value. The first step of this process can easily take between 3 and 5 min for families with < 100 individuals; hence, the overall time for one gene is directly dependent on the number of families and the time required for a genome-wide analysis is proportional to the number of genes being tested. Although it is possible to parallelize the jobs using a high-performance cluster (if available) this program is the slowest of all tested.

One of the major drawbacks we found is that some of these programs do not accept missing data (FSKAT or RareIBD) or will not generate a *p*-value if the gene set contains only one variant (GSKAT, PedGene or FarVAT). FSKAT does not accept missing data, and although it calculated *p*-values for genes that only have one informative SNP (one-SNP-gene), there were at least 75 (3.26%) of 2,154 one-SNP-genes for which the returned *p*-value was “2.” GSKAT did not provide *p*-values for more than 1,875 one-SNP-genes. PedGene also had trouble generating *p*-values for 44 one-SNP-genes out of a total of 1,916 singletons. FarVAT did not generate *p*-values for the one-SNP-genes using the Burden and SKATO models but it did generate *p*-values using the CMC and CLP models for the same 1,875 one-SNP-genes.

### Candidate genes for FASe project

Our results indicate that transmission disequilibrium tests identify genes that have a Mendelian behavior, whereas collapsing and variance-component tests identify genes that confer risk for disease. Therefore, we decided to combine and compare results from all approaches to identify the genes with most consistent results (Table 8).

**Table 8:** Most frequent genes, within *p*-value threshold category, across the different gene-based family-based methods tested^*^.

***P-value* threshold**	**gene**	**No**.	**EPACTS**			**FSKAT**	**GSKAT**		**RVGDT**	**SKAT**	**FarVAT**					**FarVAT-BLUP**					**Rare-IBD**
			**CMC**	**VT**	**SKAT**		**SKAT**	**Burden**		**IBS**	**CMC**	**CLP**	**Burden**	**CALPHA**	**SKATO**	**CMC**	**CLP**	**Burden**	**CALPHA**	**SKATO**	
≤5 × 10^−7^	*CHRD*	3	0.007	0.031	2.42 × 10^−5^	1.50 × 10^−5^	0.013	0.013	0.990	7.94 × 10^−5^	0.007	0.007	0.007	**2.09** × **10**^−7^	**3.54** × **10**^−7^	0.004	0.004	0.004	**4.06** × **10**^−7^	7.37 × 10^−7^	0.071
≤5 × 10^−6^	*CHRD*	4	0.007	0.031	0.000	0.000	0.013	0.013	0.990	0.000	0.007	0.007	0.007	**2.09** × **10**^−7^	**3.54** × **10**^−7^	0.004	0.004	0.004	**4.06** × **10**^−7^	**7.37** × **10**^−7^	0.071
≤5 × 10^−5^	*CHRD*	5	0.007	0.031	**2.42** × **10**^−5^	**1.50** × **10**^−5^	0.013	0.013	0.990	0.000	0.007	0.007	0.007	**2.09** × **10**^−7^	**3.54** × **10**^−7^	0.004	0.004	0.004	**4.06** × **10**^−7^	**7.37** × **10**^−7^	0.071
	*CLCN2*	4	0.018	0.043	2.33 × 10^−4^	2.07 × 10^−4^	0.002	0.020	1.000	7.30 × 10^−4^	0.006	0.005	0.005	**6.46** × **10**^−6^	**1.12** × **10**^−5^	0.011	0.009	0.009	**6.51** × **10**^−6^	**1.32** × **10**^−5^	0.299
	*MAS1L*	3	0.002	0.003	0.057	0.019	0.187	0.187	0.998	0.042	4.65 × 10^−4^	**1.25** × **10**^−5^	**1.25** × **10**^−5^	4.27 × 10^−4^	**1.96** × **10**^−5^	0.001	1.32 × 10^−4^	1.32 × 10^−4^	0.015	2.73 × 10^−4^	0.685
	*PTK2B*	3	0.001	0.009	0.331	0.205	0.090	0.090	1.000	0.193	1.23 × 10^−4^	**1.31** × **10**^−5^	**1.31** × **10**^−5^	0.060	**2.46** × **10**^−5^	0.001	2.39 × 10^−4^	2.39 × 10^−4^	0.113	4.93 × 10^−4^	0.443
≤5 × 10^−4^	*CPAMD8*	8	0.002	0.003	0.652	0.178	0.155	0.191	9.99 × 10^−4^	0.572	**6.91** × **10**^−5^	**2.02** × **10**^−4^	**2.02** × **10**^−4^	0.309	**4.22** × **10**^−4^	**1.69** × **10**^−4^	**2.03** × **10**^−4^	**2.03** × **10**^−4^	0.268	**4.23** × **10**^−4^	6.00 × 10^−4^
	*NLRP9*	8	0.001	0.013	0.020	0.013	0.029	0.029	0.998	0.019	**2.81** × **10**^−4^	**2.40** × **10**^−4^	**2.40** × **10**^−4^	0.002	**3.78** × **10**^−4^	**4.50** × **10**^−4^	**1.14** × **10**^−4^	**1.14** × **10**^−4^	0.003	**2.59** × **10**^−4^	0.157
	*MAS1L*	8	0.002	0.003	0.057	0.019	0.187	0.187	0.998	0.042	**4.65** × **10**^−4^	**1.25** × **10**^−5^	**1.25** × **10**^−5^	**4.27** × **10**^−4^	**1.96** × **10**^−5^	0.001	**1.32** × **10**^−4^	**1.32** × **10**^−4^	0.015	**2.73** × **10**^−4^	0.685
	*CHRD*	7	0.007	0.031	**2.42** × **10**^−5^	**1.50** × **10**^−5^	0.013	0.013	0.990	**7.94** × **10**^−5^	0.007	0.007	0.007	**2.09** × **10**^−7^	**3.54** × **10**^−7^	0.004	0.004	0.004	**4.60** × **10**^−7^	**7.37** × **10**^−7^	0.071
	*PTK2B*	7	0.001	0.009	0.331	0.205	0.090	0.090	1.000	0.193	**1.23** × **10**^−4^	**1.31** × **10**^−5^	**1.31** × **10**^−5^	0.060	**2.46** × **10**^−5^	0.001	**2.39** × **10**^−4^	**2.39** × **10**^−4^	0.113	**4.93** × **10**^−4^	0.443
	*CLCN2*	6	0.018	0.043	**2.33** × **10**^−4^	**2.07** × **10**^−4^	0.020	0.020	1.000	7.30 × 10^−4^	0.006	0.005	0.005	**6.46** × **10**^−6^	**1.12** × **10**^−5^	0.011	0.009	0.009	**6.51** × **10**^−6^	**1.32** × **10**^−5^	0.299
	*HDLBP*	5	0.002	0.024	0.009	0.001	0.031	0.032	0.996	0.002	0.021	0.028	0.028	0.068	0.046	**1.79** × **10**^−4^	**4.92** × **10**^−4^	**4.92** × **10**^−4^	**2.89** × **10**^−4^	**1.22** × **10**^−4^	0.428

*Highlighted in bold the tests with significant p-value according to threshold category*.

* *PedGene results have not been included given the inflated results of this test and the low correlation with the other gene-based methods*.

PedGene provided the most significant *p*-values for *NTN5* (Pedgene-Burden *p* = 5.80 × 10^−8^; PedGene-SKAT *p* = 1.26 × 10^−8^) and *ANKRD42* (PedGene-Burden *p* = 3.62 × 10^−7^; PedGene-SKAT *p* = 1.16 × 10^−7^). However, the inflated *p*-values observed and low correlation with any of the other software tested using the same algorithms makes us suspicious of the validity of these results.

*CHRD* was the gene with the third most significant *p*-value. *CHRD* had a *p* ≤ 5 × 10^−7^ in three different models (FarVAT-CALPHA, FarVAT-SKATO, and FarVAT-BLUP-CALPHA). Additionally, as we lowered the considered *p*-value threshold, we found that more tests identified *CHRD* as a potential candidate gene associated with AD. When we lowered the threshold to suggestive genome-wide *p*-value (*p* ≤ 5 × 10^−4^) we found that seven different models identified *CHRD* as significantly associated with AD. Following the same method we found that *CLCN2, MAS1L*, and *PTK2B* had *p* ≤ 5 × 10^−05^ in at least three tests, and if we lowered the threshold to ≤ 5 × 10^−4^ *p*-value, these genes were identified as significant by at least three additional tests.

Among genes with a *p* ≤ 5 × 10^−04^; *CPAMD8* was identified by at least nine gene-based methods (FarVAT, FarVAT-BLUP, and PedGene). The exact *p*-value for *CPAMD8* could not be estimated by RVGDT as it reported a *p*-value of 9 × 10^−04^, which is the most significant *p*-value reported by this test. Therefore, we cannot conclude that *CPAMD8* presented a *p*-value ≤ 5 × 10^−04^ by RVGDT. *CHRD, CLCN2, MAS1L, PTK2B*, and *CPAMD8, NLRP9*, and *HDLBP* were also potential novel candidate genes for familial LOAD as they had *p* ≤ 5 × 10^−04^ using at least five or more tests (Table 8).

Since these were identified by multiple gene-based methods, we wanted to determine whether any of these seven candidate genes are involved in known AD pathways. Common variants in *PTK2B* have been associated with AD risk at a genome-wide level (Lambert et al., 2013). Our results indicate there are additional low-frequency and rare nonsynonymous variants in *PTK2B* that are associated with AD risk in late-onset families.

**Figure 5:**
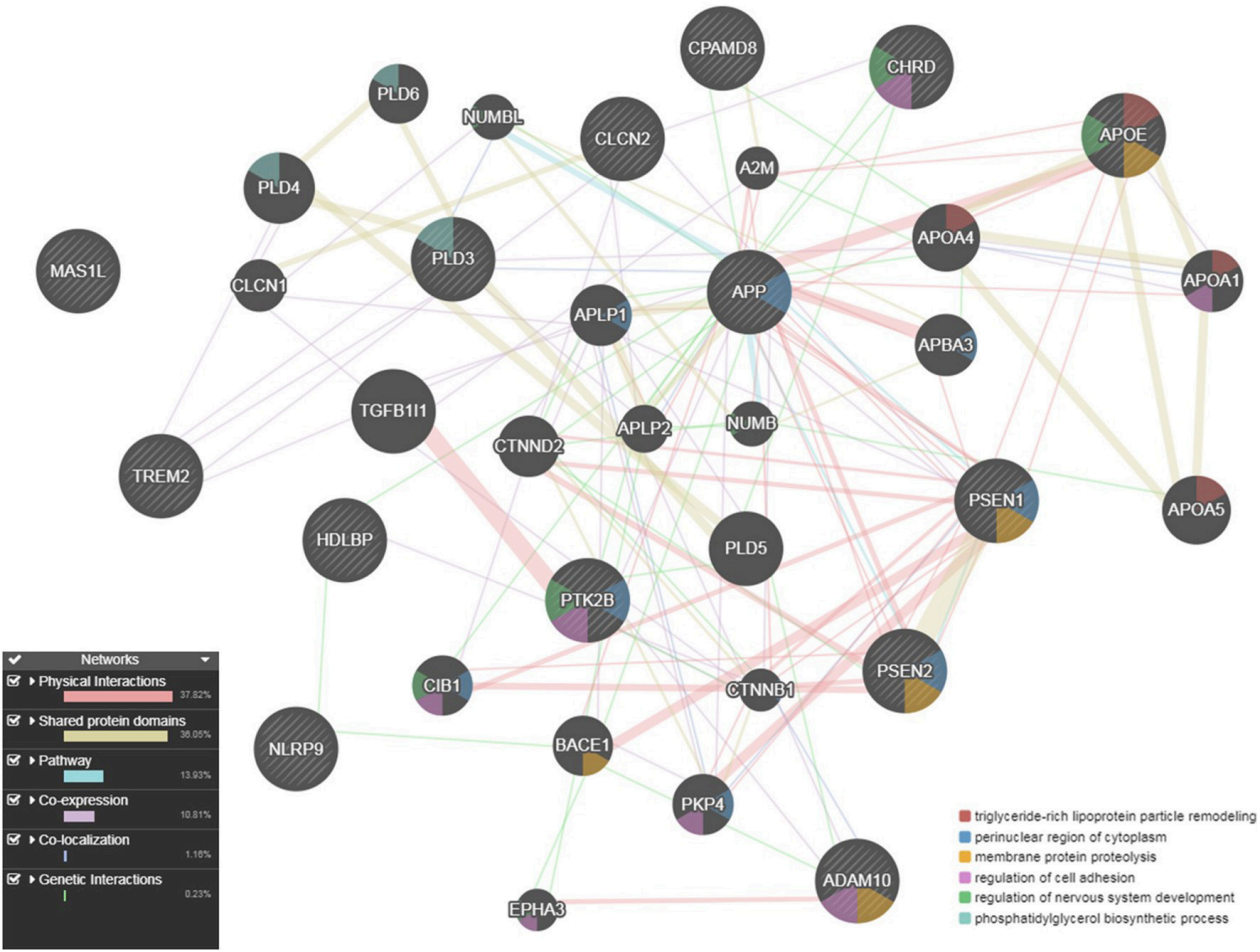
Gene network for the seven candidate genes (*CHRD, CLCN2, CPAMD8, HDLBP, MAS1L, NLRP9*, and *PTK2B*) with multiple evidence of a *p* ≤ 5 × 10^−04^, anchored with known AD genes (*APP, PSEN1, PSEN2, APOE, TREM2, ADAM10*, and *PLD3*), as described by GeneMANIA.

We used the GeneMANIA (http://pages.genemania.org/) algorithm on the seven candidate genes (*CHRD, MAS1L, PTK2B, CPAMD8, NLRP9, CLCN2*, and *HDLBP*) and known AD-related genes (*APP, PSEN1, PSEN2, APOE, TREM2, PLD3*, and *ADAM10*) which are involved in some pathways important in AD (APP-metabolism and immune response). GeneMANIA looks for relationships among a list of given genes by searching within multiple publicly available biological datasets. These datasets include protein-protein, protein-DNA and genetic interactions, pathways, reactions, gene and protein expression data, protein domains and phenotypic screening profiles. We found that our candidate genes have genetic interactions and co-localization with known AD genes. *CHRD* and *PTK2B* are involved in “regulation of cell adhesion” like *ADAM10*; *PTK2B* is involved in “regulation of neurogenesis” like *APOE* and “perinuclear region of cytoplasm” like *APP, PSEN1* and *PSEN2*. Finally, *CLCN2* and *PTK2B* are connected through “regulation of ion transport” (Figure 5).

## Discussion

The missing heritability in AD, and in many complex diseases, may be found in very rare variants for which discovery will require either large datasets (e.g., the ADSP Discovery Phase which has over 10,000 sequenced individuals) or datasets enriched for rare variants (such as families with history of AD). In this study, we present the most comprehensive performance analyses of multiple gene-based methods using 285 families with AD. Some of the current methods available are underpowered or too restrictive to detect genes significantly associated with this disease (Figure 4). Results from our simulated data (Table 5) show that only certain highly-restricted scenarios provide gene-wide significant *p*-values in family-based analyses; whereas similar scenarios in a case-control study would result in gene-wide *p*-values. To circumvent this power issue, we relied on the combination of multiple evidence toward the same gene.

One key aspect to adapt gene-based analyses to a family-based context is to account for population stratification and hidden relatedness that may appear due to the inherent nature of family datasets. To take into account this issue, gene-based algorithms must incorporate kinship matrices to model the relationships among samples. Therefore, an appropriate estimate of the kinship matrix is of utmost importance. In this work we show how different relationship matrices influence results. We tested the three most common types of kinship matrix, pedigree reconstruction (PED), identity by state (IBS), and Balding-Nichols (BN). We show that for a situation of complex incomplete families, correction using PED or BN matrices will lead to an overcorrection of the relationships decreasing the power of these tests (Table 7, Figure 4A).

In order to choose the best gene-based algorithm for analysis, it is important to take into account the nature (impact and directionality) of the variants that are being included in the test. Collapsing tests are powerful when a large proportion of variants are causal and the effects are in the same direction. Variance-component tests are supposed to be more powerful than collapsing tests because they allow for admixture of risk and protective variants within the region being tested (Ionita-Laza et al., 2013). It is not practical to account for the nature of the variants included in each gene-set, and the true disease model is unknown and variable; hence, omnibus or combined tests such as SKAT-O would be desirable for genome-wide studies (Lee et al., 2012). However, most family-based methods do not incorporate the SKAT-O algorithm, except FarVAT. Therefore, the best approach to perform genome-wide rare variant discovery is to combine different algorithms and look for common signatures across the tests performed. Nonetheless, we are aware that running all available tests is a time-consuming task that requires additional expertise and resources. In our analyses FarVAT, with the BLUP adjustment, provide the best results in terms of significant *p*-values and minor inflation, for genome-wide gene-based analysis; it is a fast software that provides results from multiple tests at the same time. The R version of SKAT or EPACTS would be alternatively valid choices, taking into account that these overcorrect and the *p*-value threshold should be lowered.

In this study, we identified *CHRD* as a candidate gene with a genome-wide significant *p*-value (5 × 10^−07^) reported by three tests, and another six genes that had a suggestive genome-wide *p* < 5 × 10^−04^ in at least five, and up to nine, of the different test performed: *CLCN2, CPAMD8, HDLBP, MAS1L, NLRP9*, and *PTK2B*. Additionally, these genes seem to have direct and indirect interactions (genetic interaction, co-localization or shared function) with known AD genes (*APP, PSEN1, PSEN2, APOE, TREM2, PLD3*, and *ADAM10*).

*CHRD*, chordin, is a highly-conserved developmental protein which inhibits the ventralizing activity of bone morphogenetic proteins, is active during gastrulation, expressed in fetal and adult liver and cerebellum, and is associated with Cornelia de Lange syndrome (Smith et al., 1999). *CLCN2*, chloride voltage-gated channel 2, has several functions including the regulation of cell volume: membrane potential stabilization, signal transduction and transepithelial transport. It has been associated with different epilepsy modes (Saint-Martin et al., 2009; Cukier et al., 2014) and leukoencephalopathy (Gaitán-Peñas et al., 2017). *CHRD* and *CLCN2* show co-expression which could be due to their close proximity, both belong to a gene cluster at 3q27. Interestingly, *CLCN2* shows co-expression with *TREM2* which, other than being an AD risk gene, is known to cause leukoencephalopathy in PLOSL (polycystic lipomembranous osteodysplasia with sclerosing leukoencephalopathy), also known as Nasu-Hakola disease.

*PTK2B*, Protein Tyrosine Kinase 2 Beta, was described as an AD risk locus in the largest GWAS meta-analysis conducted to date (Lambert et al., 2013), and later corroborated by others (Beecham et al., 2014; Wang et al., 2015). The protein encoded by *PTK2B* is a member of the focal adhesion kinase (FAK) family that can be activated by changes in intracellular calcium levels, which are disrupted in AD brains. Its activation regulates neuronal activity such as mitogen-activated protein kinase (MAPK) signaling (Rosenthal and Kamboh, 2014). *PTK2B* could also be involved in hippocampal synaptic function (Lambert et al., 2013). Although there is no co-expression or genetic interaction between *CLCN2* and *PTK2B*, both are involved in regulation of ion transport. Additionally, *PTK2B* is involved in regulation of lipidic metabolic processes like *APOE*, a cholesterol-related gene. Although no association has yet been reported between *APOE* and *HDLBP*, the High-Density Lipoprotein Binding Protein, the latter plays a role in cell sterol metabolism, protecting cells from over-accumulation of cholesterol, which has been reported as risk factor for atherosclerotic vascular diseases.

*CPAMD8*, C3 and PZP Like, Alpha-2-Macroglobulin Domain Containing 8, has been previously associated with neurological conditions other than AD. Common variants in *CPAMD8* were found among top markers associated with multiple sclerosis (Baranzini et al., 2009). Missense and frameshift variants in *CPAMD8* were identified in three families affected with Anterior Segment Dysgenesis (Cheong et al., 2016). According to the UKBiobank PheWeb (http://pheweb.sph.umich.edu:5000/), *CPAMD8* has a 2.9 × 10^−9^ *p*-value for its association with AD. We did not find any shared pathway between *CPAMD8* and known AD genes in the GeneMANIA network, even though it seems to have a genetic interaction with *APP* (Lin et al., 2010). In our study *CPAMD8* was identified as a candidate gene (with *p* < 1 × 10^−4^) for AD by at least nine gene-based methods from different software, and we found that several variants within this gene had varying degrees of segregation in more than twenty families. Variant p.(Ser1103Ala) segregates with disease status in two families with two and three carriers respectively, and is present in another two families. Variant p.(His465Arg) segregates with disease status in five families with two or three carriers per family and is present in another 11 families. Variant p.(Arg1380Cys) is private to a family with three carriers, p.(Ala1492Pro) is private to a family with five carriers, and p.(Val521Met) is private to a family with three carriers.

*MAS1L*, MAS1 Proto-Oncogene Like, is a G Protein-Coupled Receptor. Members of this family of membrane proteins are activated by a wide spectrum of ligands and modulate the activity of different signaling pathways in a ligand-specific manner. Aly et al. (2008) described polymorphisms in the region of the UBD/MAS1L genes that are associated with type-1 diabetes.

The immune system and the integrity of the blood-brain barrier are key factors for Alzheimer disease. *NLRP9*, NLR Family Pyrin Domain Containing 9, has been involved in inflammation response. Nyúl-Tóth et al. (2017) found *NLRP9* expressed in cerebral endothelial cells and, at much lower levels, in brain pericytes; and another member of the NLP family (*NLRP1*) has been associated with AD (Pontillo et al., 2012).

We have reviewed more than 22 algorithms from eight different software available for gene-based analyses in complex families. After a thorough examination of the performance of these tests under different scenarios, we present a methodology to identify genes associated with the studied phenotype. We have applied this methodology to 285 European-American families affected with late onset Alzheimer disease (LOAD) and we identified six candidate genes with suggestive or genome-wide significant *p*-values across different software and algorithms. Based on the consistency of our results, we are confident that some of these genes may play a role in AD pathology and therefore are of interest to follow up in replication and functional studies.

## Author contributions

MF performed processing and quality control of data, implementation of software and statistical analysis; participated in study design, interpretation of results and wrote the manuscript. JB contributed to data collection, processing and quality control. JB, JD-A, LI, YD, and OH contributed on software implementation and interpretation or results. JN, JM, and AG contributed to study participant recruitment and sample collection. CC collected data, participated in study design, interpretation of results and revision of manuscript. All the authors read and provided input to the manuscript.

### Conflict of interest statement

The authors declare that the research was conducted in the absence of any commercial or financial relationships that could be construed as a potential conflict of interest.

## Supplementary material

Figure S1 Schematic representation of the bioinformatics pipeline used in this study to filter and combine the information from whole exome sequencing (WES) and whole genome sequencing (WGS).

Table S1 Structure of the families used in this study with detail of the number of individuals (IDs) sequenced per family, number of cases (CA), number of controls (CO), number of females (Fe) and number of males (Ma). The first 25 families were employed in the simulation analysis.

Table S2 Design of simulated “GENE-A” across 25 families. Scenarios 5FCx0FNC, 5FCx5FNC, 5FCx10FNC, 5FCx15FNC, 5FCx20FNC.

Table S3 Comparison of kinship matrices for Fam#1 and Fam#2.
