# Supplementary figures and images for: Evaluation of Gene-Based Family-Based Methods to Detect Novel Genes Associated With Familial Late Onset Alzheimer Disease
Source: Front Neurosci. 2018 Apr 4;12:209. doi: 10.3389/fnins.2018.00209 (PMC5893779; doi:10.3389/fnins.2018.00209)

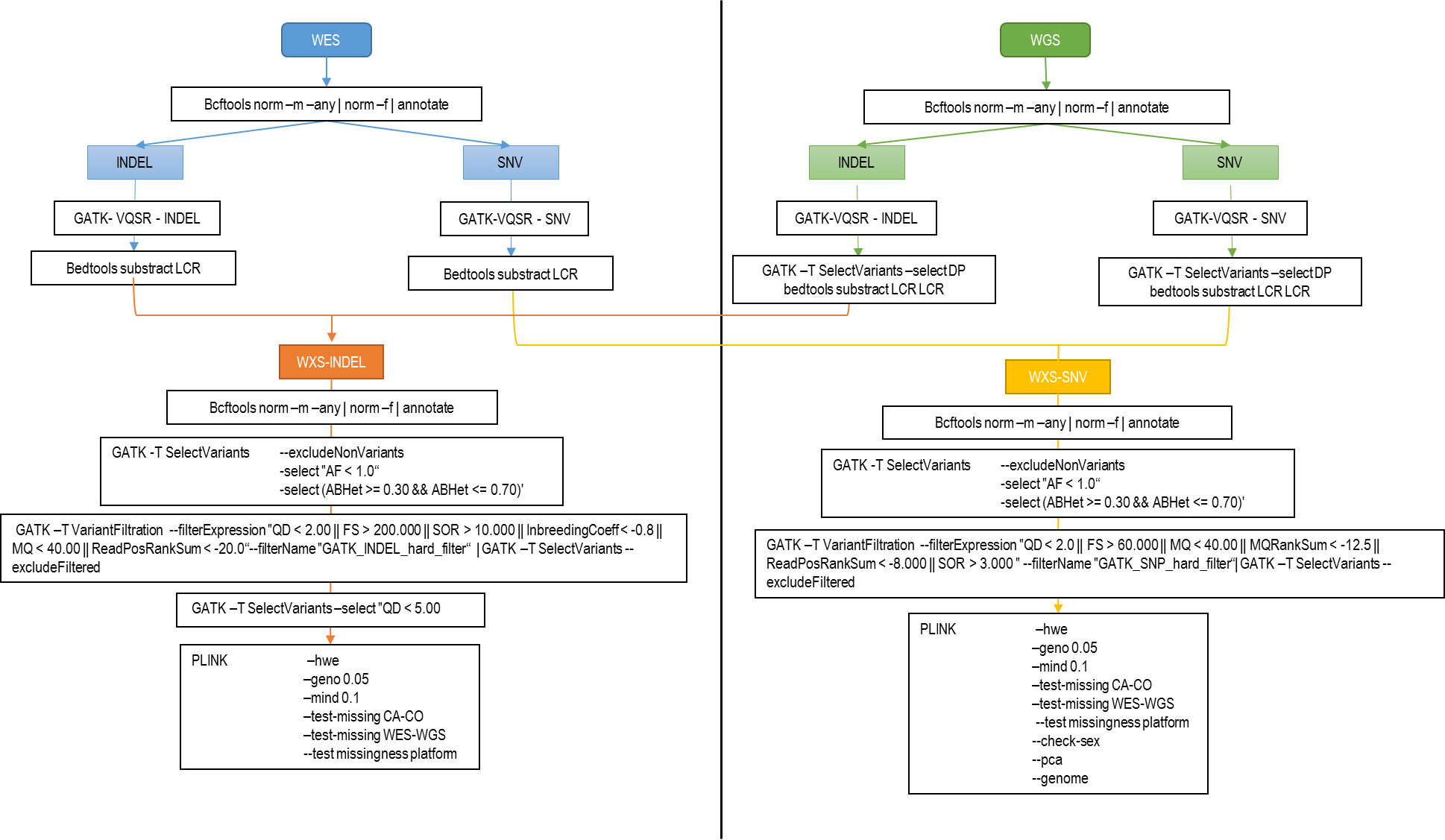

Supplement: Figure S1 — Schematic representation of the bioinformatics pipeline used in this study to filter and combine the information from whole exome sequencing (WES) and whole genome sequencing (WGS). [file Image1.PNG]
