# Supplementary material for: Evaluation of Gene-Based Family-Based Methods to Detect Novel Genes Associated With Familial Late Onset Alzheimer Disease
Source: Front Neurosci. 2018 Apr 4;12:209. doi: 10.3389/fnins.2018.00209 (PMC5893779; doi:10.3389/fnins.2018.00209)
Supplement: Table S1 — Structure of the families used in this study with detail of the number of individuals (IDs) sequenced per family, number of cases (CA), number of controls (CO), number of females (Fe) and number of males (Ma). The first 25 families were employed in the simulation analysis. [file Table1.DOCX]

**Table S1.** Structure of the families used in this study with detail of the number of individuals (IDs) sequenced per family, number of cases (CA), number of controls (CO), number of females (Fe) and number of males (Ma).The first 25 families were employed din the simulation analysis.

| Fam # | IDs/fam | CA/fam | CO/fam | Fe | Ma |
| --- | --- | --- | --- | --- | --- |
| Fam1 | 3 | 2 | 1 | 3 | 0 |
| Fam2 | 4 | 3 | 1 | 4 | 0 |
| Fam3 | 5 | 4 | 1 | 4 | 1 |
| Fam4 | 6 | 4 | 2 | 3 | 3 |
| Fam5 | 7 | 5 | 2 | 6 | 1 |
| Fam6 | 3 | 2 | 1 | 3 | 0 |
| Fam7 | 4 | 3 | 1 | 4 | 0 |
| Fam8 | 5 | 4 | 1 | 4 | 1 |
| Fam9 | 6 | 5 | 1 | 4 | 2 |
| Fam10 | 7 | 5 | 2 | 3 | 4 |
| Fam11 | 3 | 2 | 1 | 3 | 0 |
| Fam12 | 4 | 3 | 1 | 2 | 2 |
| Fam13 | 5 | 4 | 1 | 4 | 1 |
| Fam14 | 6 | 5 | 1 | 5 | 1 |
| Fam15 | 7 | 6 | 1 | 6 | 1 |
| Fam16 | 3 | 2 | 1 | 2 | 1 |
| Fam17 | 4 | 3 | 1 | 3 | 1 |
| Fam18 | 5 | 4 | 1 | 4 | 1 |
| Fam19 | 6 | 5 | 1 | 4 | 2 |
| Fam20 | 7 | 5 | 2 | 3 | 4 |
| Fam21 | 3 | 2 | 1 | 1 | 2 |
| Fam22 | 4 | 3 | 1 | 4 | 0 |
| Fam23 | 5 | 4 | 1 | 3 | 2 |
| Fam24 | 6 | 4 | 2 | 3 | 3 |
| Fam25 | 7 | 5 | 2 | 5 | 2 |
| Fam26 | 2 | 1 | 1 | 2 | 0 |
| Fam27 | 2 | 1 | 1 | 2 | 0 |
| Fam28 | 2 | 1 | 1 | 1 | 1 |
| Fam29 | 2 | 1 | 1 | 1 | 1 |
| Fam30 | 2 | 1 | 1 | 2 | 0 |
| Fam31 | 2 | 1 | 1 | 1 | 1 |
| Fam32 | 2 | 1 | 1 | 1 | 1 |
| Fam33 | 2 | 1 | 1 | 1 | 1 |
| Fam34 | 3 | 2 | 1 | 2 | 1 |
| Fam35 | 3 | 1 | 2 | 3 | 0 |
| Fam36 | 3 | 2 | 1 | 3 | 0 |
| Fam37 | 3 | 2 | 1 | 3 | 0 |
| Fam38 | 3 | 2 | 1 | 2 | 1 |
| Fam39 | 3 | 2 | 1 | 3 | 0 |
| Fam40 | 3 | 2 | 1 | 1 | 2 |
| Fam41 | 3 | 2 | 1 | 1 | 2 |
| Fam42 | 3 | 2 | 1 | 2 | 1 |
| Fam43 | 3 | 2 | 1 | 2 | 1 |
| Fam44 | 3 | 2 | 1 | 2 | 1 |
| Fam45 | 3 | 2 | 1 | 2 | 1 |
| Fam46 | 3 | 1 | 2 | 2 | 1 |
| Fam47 | 3 | 2 | 1 | 2 | 1 |
| Fam48 | 3 | 2 | 1 | 3 | 0 |
| Fam49 | 3 | 2 | 1 | 3 | 0 |
| Fam50 | 3 | 2 | 1 | 1 | 2 |
| Fam51 | 3 | 2 | 1 | 1 | 2 |
| Fam52 | 3 | 1 | 2 | 1 | 2 |
| Fam53 | 3 | 2 | 1 | 2 | 1 |
| Fam54 | 3 | 2 | 1 | 1 | 2 |
| Fam55 | 3 | 2 | 1 | 3 | 0 |
| Fam56 | 3 | 2 | 1 | 1 | 2 |
| Fam57 | 3 | 2 | 1 | 2 | 1 |
| Fam58 | 3 | 2 | 1 | 1 | 2 |
| Fam59 | 3 | 2 | 1 | 1 | 2 |
| Fam60 | 3 | 2 | 1 | 3 | 0 |
| Fam61 | 3 | 2 | 1 | 1 | 2 |
| Fam62 | 3 | 2 | 1 | 3 | 0 |
| Fam63 | 3 | 2 | 1 | 1 | 2 |
| Fam64 | 3 | 2 | 1 | 2 | 1 |
| Fam65 | 3 | 2 | 1 | 1 | 2 |
| Fam66 | 3 | 2 | 1 | 2 | 1 |
| Fam67 | 3 | 1 | 2 | 3 | 0 |
| Fam68 | 3 | 2 | 1 | 1 | 2 |
| Fam69 | 3 | 1 | 2 | 2 | 1 |
| Fam70 | 3 | 2 | 1 | 1 | 2 |
| Fam71 | 3 | 2 | 1 | 1 | 2 |
| Fam72 | 3 | 2 | 1 | 0 | 3 |
| Fam73 | 3 | 2 | 1 | 0 | 3 |
| Fam74 | 3 | 2 | 1 | 2 | 1 |
| Fam75 | 3 | 2 | 1 | 2 | 1 |
| Fam76 | 3 | 2 | 1 | 3 | 0 |
| Fam77 | 3 | 2 | 1 | 1 | 2 |
| Fam78 | 3 | 2 | 1 | 0 | 3 |
| Fam79 | 3 | 2 | 1 | 1 | 2 |
| Fam80 | 3 | 2 | 1 | 0 | 3 |
| Fam81 | 3 | 2 | 1 | 3 | 0 |
| Fam82 | 3 | 2 | 1 | 2 | 1 |
| Fam83 | 3 | 2 | 1 | 1 | 2 |
| Fam84 | 3 | 2 | 1 | 2 | 1 |
| Fam85 | 3 | 2 | 1 | 1 | 2 |
| Fam86 | 3 | 2 | 1 | 0 | 3 |
| Fam87 | 3 | 1 | 2 | 2 | 1 |
| Fam88 | 3 | 2 | 1 | 2 | 1 |
| Fam89 | 3 | 2 | 1 | 1 | 2 |
| Fam90 | 3 | 2 | 1 | 1 | 2 |
| Fam91 | 3 | 2 | 1 | 1 | 2 |
| Fam92 | 3 | 2 | 1 | 2 | 1 |
| Fam93 | 3 | 2 | 1 | 3 | 0 |
| Fam94 | 3 | 2 | 1 | 1 | 2 |
| Fam95 | 3 | 2 | 1 | 1 | 2 |
| Fam96 | 3 | 2 | 1 | 3 | 0 |
| Fam97 | 3 | 2 | 1 | 2 | 1 |
| Fam98 | 3 | 2 | 1 | 1 | 2 |
| Fam99 | 3 | 2 | 1 | 3 | 0 |
| Fam100 | 3 | 2 | 1 | 3 | 0 |
| Fam101 | 3 | 2 | 1 | 1 | 2 |
| Fam102 | 4 | 3 | 1 | 3 | 1 |
| Fam103 | 4 | 2 | 2 | 2 | 2 |
| Fam104 | 4 | 2 | 2 | 0 | 4 |
| Fam105 | 4 | 2 | 2 | 2 | 2 |
| Fam106 | 4 | 3 | 1 | 4 | 0 |
| Fam107 | 4 | 2 | 2 | 3 | 1 |
| Fam108 | 4 | 3 | 1 | 1 | 3 |
| Fam109 | 4 | 2 | 2 | 3 | 1 |
| Fam110 | 4 | 2 | 2 | 2 | 2 |
| Fam111 | 4 | 2 | 2 | 2 | 2 |
| Fam112 | 4 | 2 | 2 | 4 | 0 |
| Fam113 | 4 | 2 | 2 | 4 | 0 |
| Fam114 | 4 | 3 | 1 | 2 | 2 |
| Fam115 | 4 | 3 | 1 | 3 | 1 |
| Fam116 | 4 | 3 | 1 | 3 | 1 |
| Fam117 | 4 | 3 | 1 | 4 | 0 |
| Fam118 | 4 | 2 | 2 | 3 | 1 |
| Fam119 | 4 | 2 | 2 | 1 | 3 |
| Fam120 | 4 | 2 | 2 | 3 | 1 |
| Fam121 | 4 | 3 | 1 | 3 | 1 |
| Fam122 | 4 | 3 | 1 | 2 | 2 |
| Fam123 | 4 | 3 | 1 | 2 | 2 |
| Fam124 | 4 | 3 | 1 | 3 | 1 |
| Fam125 | 4 | 2 | 2 | 3 | 1 |
| Fam126 | 4 | 3 | 1 | 4 | 0 |
| Fam127 | 4 | 3 | 1 | 2 | 2 |
| Fam128 | 4 | 2 | 2 | 2 | 2 |
| Fam129 | 4 | 2 | 2 | 3 | 1 |
| Fam130 | 4 | 2 | 2 | 3 | 1 |
| Fam131 | 4 | 2 | 2 | 1 | 3 |
| Fam132 | 4 | 2 | 2 | 3 | 1 |
| Fam133 | 4 | 2 | 2 | 3 | 1 |
| Fam134 | 4 | 3 | 1 | 2 | 2 |
| Fam135 | 4 | 3 | 1 | 2 | 2 |
| Fam136 | 4 | 3 | 1 | 2 | 2 |
| Fam137 | 4 | 2 | 2 | 3 | 1 |
| Fam138 | 4 | 3 | 1 | 3 | 1 |
| Fam139 | 4 | 3 | 1 | 3 | 1 |
| Fam140 | 4 | 3 | 1 | 4 | 0 |
| Fam141 | 4 | 2 | 2 | 2 | 2 |
| Fam142 | 4 | 2 | 2 | 4 | 0 |
| Fam143 | 4 | 3 | 1 | 3 | 1 |
| Fam144 | 4 | 2 | 2 | 1 | 3 |
| Fam145 | 4 | 3 | 1 | 2 | 2 |
| Fam146 | 4 | 3 | 1 | 1 | 3 |
| Fam147 | 4 | 3 | 1 | 3 | 1 |
| Fam148 | 4 | 3 | 1 | 3 | 1 |
| Fam149 | 4 | 3 | 1 | 3 | 1 |
| Fam150 | 4 | 3 | 1 | 3 | 1 |
| Fam151 | 4 | 2 | 2 | 3 | 1 |
| Fam152 | 4 | 2 | 2 | 1 | 3 |
| Fam153 | 4 | 3 | 1 | 4 | 0 |
| Fam154 | 4 | 2 | 2 | 2 | 2 |
| Fam155 | 4 | 2 | 2 | 2 | 2 |
| Fam156 | 4 | 2 | 2 | 2 | 2 |
| Fam157 | 4 | 2 | 2 | 2 | 2 |
| Fam158 | 4 | 3 | 1 | 2 | 2 |
| Fam159 | 4 | 3 | 1 | 4 | 0 |
| Fam160 | 4 | 2 | 2 | 2 | 2 |
| Fam161 | 4 | 2 | 2 | 1 | 3 |
| Fam162 | 4 | 3 | 1 | 2 | 2 |
| Fam163 | 4 | 2 | 2 | 2 | 2 |
| Fam164 | 4 | 2 | 2 | 1 | 3 |
| Fam165 | 4 | 3 | 1 | 1 | 3 |
| Fam166 | 4 | 2 | 2 | 3 | 1 |
| Fam167 | 4 | 3 | 1 | 4 | 0 |
| Fam168 | 4 | 2 | 2 | 3 | 1 |
| Fam169 | 4 | 2 | 2 | 2 | 2 |
| Fam170 | 4 | 3 | 1 | 2 | 2 |
| Fam171 | 4 | 3 | 1 | 2 | 2 |
| Fam172 | 4 | 3 | 1 | 2 | 2 |
| Fam173 | 4 | 2 | 2 | 3 | 1 |
| Fam174 | 4 | 2 | 2 | 2 | 2 |
| Fam175 | 4 | 2 | 2 | 4 | 0 |
| Fam176 | 4 | 2 | 2 | 3 | 1 |
| Fam177 | 4 | 2 | 2 | 2 | 2 |
| Fam178 | 4 | 2 | 2 | 2 | 2 |
| Fam179 | 4 | 3 | 1 | 1 | 3 |
| Fam180 | 4 | 2 | 2 | 1 | 3 |
| Fam181 | 4 | 3 | 1 | 4 | 0 |
| Fam182 | 4 | 3 | 1 | 2 | 2 |
| Fam183 | 4 | 3 | 1 | 1 | 3 |
| Fam184 | 4 | 2 | 2 | 3 | 1 |
| Fam185 | 4 | 3 | 1 | 3 | 1 |
| Fam186 | 4 | 3 | 1 | 2 | 2 |
| Fam187 | 4 | 3 | 1 | 3 | 1 |
| Fam188 | 4 | 3 | 1 | 1 | 3 |
| Fam189 | 4 | 3 | 1 | 0 | 4 |
| Fam190 | 4 | 3 | 1 | 2 | 2 |
| Fam191 | 4 | 3 | 1 | 1 | 3 |
| Fam192 | 4 | 2 | 2 | 3 | 1 |
| Fam193 | 4 | 2 | 2 | 2 | 2 |
| Fam194 | 4 | 2 | 2 | 4 | 0 |
| Fam195 | 4 | 2 | 2 | 2 | 2 |
| Fam196 | 5 | 3 | 2 | 3 | 2 |
| Fam197 | 5 | 3 | 2 | 2 | 3 |
| Fam198 | 5 | 4 | 1 | 2 | 3 |
| Fam199 | 5 | 4 | 1 | 2 | 3 |
| Fam200 | 5 | 2 | 3 | 5 | 0 |
| Fam201 | 5 | 3 | 2 | 4 | 1 |
| Fam202 | 5 | 3 | 2 | 2 | 3 |
| Fam203 | 5 | 4 | 1 | 4 | 1 |
| Fam204 | 5 | 3 | 2 | 1 | 4 |
| Fam205 | 5 | 3 | 2 | 4 | 1 |
| Fam206 | 5 | 4 | 1 | 5 | 0 |
| Fam207 | 5 | 3 | 2 | 2 | 3 |
| Fam208 | 5 | 3 | 2 | 3 | 2 |
| Fam209 | 5 | 3 | 2 | 1 | 4 |
| Fam210 | 5 | 3 | 2 | 2 | 3 |
| Fam211 | 5 | 4 | 1 | 5 | 0 |
| Fam212 | 5 | 4 | 1 | 3 | 2 |
| Fam213 | 5 | 3 | 2 | 4 | 1 |
| Fam214 | 5 | 3 | 2 | 4 | 1 |
| Fam215 | 5 | 3 | 2 | 4 | 1 |
| Fam216 | 5 | 4 | 1 | 5 | 0 |
| Fam217 | 5 | 3 | 2 | 3 | 2 |
| Fam218 | 5 | 4 | 1 | 5 | 0 |
| Fam219 | 5 | 3 | 2 | 3 | 2 |
| Fam220 | 5 | 3 | 2 | 2 | 3 |
| Fam221 | 5 | 3 | 2 | 5 | 0 |
| Fam222 | 5 | 4 | 1 | 3 | 2 |
| Fam223 | 5 | 3 | 2 | 5 | 0 |
| Fam224 | 5 | 4 | 1 | 1 | 4 |
| Fam225 | 5 | 3 | 2 | 4 | 1 |
| Fam226 | 5 | 3 | 2 | 1 | 4 |
| Fam227 | 5 | 3 | 2 | 4 | 1 |
| Fam228 | 5 | 4 | 1 | 4 | 1 |
| Fam229 | 5 | 4 | 1 | 2 | 3 |
| Fam230 | 5 | 4 | 1 | 1 | 4 |
| Fam231 | 5 | 3 | 2 | 5 | 0 |
| Fam232 | 5 | 2 | 3 | 4 | 1 |
| Fam233 | 5 | 4 | 1 | 3 | 2 |
| Fam234 | 5 | 4 | 1 | 3 | 2 |
| Fam235 | 5 | 4 | 1 | 3 | 2 |
| Fam236 | 5 | 4 | 1 | 4 | 1 |
| Fam237 | 5 | 3 | 2 | 4 | 1 |
| Fam238 | 5 | 4 | 1 | 3 | 2 |
| Fam239 | 5 | 4 | 1 | 2 | 3 |
| Fam240 | 5 | 4 | 1 | 1 | 4 |
| Fam241 | 5 | 3 | 2 | 2 | 3 |
| Fam242 | 5 | 3 | 2 | 1 | 4 |
| Fam243 | 5 | 3 | 2 | 3 | 2 |
| Fam244 | 5 | 3 | 2 | 2 | 3 |
| Fam245 | 5 | 3 | 2 | 3 | 2 |
| Fam246 | 5 | 4 | 1 | 1 | 4 |
| Fam247 | 5 | 4 | 1 | 2 | 3 |
| Fam248 | 6 | 4 | 2 | 5 | 1 |
| Fam249 | 6 | 5 | 1 | 4 | 2 |
| Fam250 | 6 | 5 | 1 | 4 | 2 |
| Fam251 | 6 | 5 | 1 | 4 | 2 |
| Fam252 | 6 | 4 | 2 | 3 | 3 |
| Fam253 | 6 | 4 | 2 | 4 | 2 |
| Fam254 | 6 | 5 | 1 | 6 | 0 |
| Fam255 | 6 | 4 | 2 | 2 | 4 |
| Fam256 | 6 | 4 | 2 | 5 | 1 |
| Fam257 | 6 | 4 | 2 | 4 | 2 |
| Fam258 | 6 | 4 | 2 | 5 | 1 |
| Fam259 | 6 | 5 | 1 | 5 | 1 |
| Fam260 | 6 | 5 | 1 | 5 | 1 |
| Fam261 | 6 | 4 | 2 | 3 | 3 |
| Fam262 | 6 | 4 | 2 | 3 | 3 |
| Fam263 | 6 | 4 | 2 | 4 | 2 |
| Fam264 | 6 | 5 | 1 | 4 | 2 |
| Fam265 | 6 | 4 | 2 | 4 | 2 |
| Fam266 | 6 | 3 | 3 | 4 | 2 |
| Fam267 | 6 | 4 | 2 | 2 | 4 |
| Fam268 | 7 | 5 | 2 | 4 | 3 |
| Fam269 | 7 | 6 | 1 | 5 | 2 |
| Fam270 | 7 | 4 | 3 | 6 | 1 |
| Fam271 | 7 | 4 | 3 | 4 | 3 |
| Fam272 | 7 | 5 | 2 | 6 | 1 |
| Fam273 | 7 | 5 | 2 | 2 | 5 |
| Fam274 | 7 | 5 | 2 | 6 | 1 |
| Fam275 | 7 | 5 | 2 | 2 | 5 |
| Fam276 | 7 | 5 | 2 | 4 | 3 |
| Fam277 | 7 | 6 | 1 | 4 | 3 |
| Fam278 | 7 | 3 | 4 | 1 | 6 |
| Fam279 | 7 | 5 | 2 | 4 | 3 |
| Fam280 | 7 | 5 | 2 | 3 | 4 |
| Fam281 | 8 | 6 | 2 | 4 | 4 |
| Fam282 | 8 | 6 | 2 | 4 | 4 |
| Fam283 | 9 | 8 | 1 | 8 | 1 |
| Fam284 | 9 | 8 | 1 | 6 | 3 |
| Fam285 | 9 | 5 | 4 | 6 | 3 |
