# Supplementary material for: Evaluation of Gene-Based Family-Based Methods to Detect Novel Genes Associated With Familial Late Onset Alzheimer Disease
Source: Front Neurosci. 2018 Apr 4;12:209. doi: 10.3389/fnins.2018.00209 (PMC5893779; doi:10.3389/fnins.2018.00209)
Supplement: Table S2 — Design of simulated “GENE-A” across 25 families. Scenarios 5FCx0FNC, 5FCx5FNC, 5FCx10FNC, 5FCx15FNC, 5FCx20FNC. [file Table2.DOCX]

**Table S2**. Design of simulated “GENE-A” across 25 families. Scenarios 5FCx0FNC, 5FCx5FNC, 5FCx10FNC, 5FCx15FNC, 5FCx20FNC.

|  | | | | | |  | 5FCx0FNC | | | | |  | 5FCx5FNC | | | | |  | 5FCx10FNC | | | | |  | 5FCx15FNC | | | | |  | 5FCx20FNC | | | | |
| --- | --- | --- | --- | --- | --- | --- | --- | --- | --- | --- | --- | --- | --- | --- | --- | --- | --- | --- | --- | --- | --- | --- | --- | --- | --- | --- | --- | --- | --- | --- | --- | --- | --- | --- | --- |
| FID | IID | PID | MID | SEX | STATUS |  | a1 | a2 | a3 | a4 | a5 |  | a1 | a2 | a3 | a4 | a5 |  | a1 | a2 | a3 | a4 | a5 |  | a1 | a2 | a3 | a4 | a5 |  | a1 | a2 | a3 | a4 | a5 |
| FAM1 | FAM1_1 | FAM1_5 | FAM1_4 | 2 | 2 |  | 1 | 0 | 0 | 0 | 0 |  | 1 | 0 | 0 | 0 | 0 |  | 1 | 0 | 0 | 0 | 0 |  | 1 | 0 | 0 | 0 | 0 |  | 1 | 0 | 0 | 0 | 0 |
| FAM1 | FAM1_7 | FAM1_5 | FAM1_4 | 1 | 1 |  | 0 | 0 | 0 | 0 | 0 |  | 0 | 0 | 0 | 0 | 0 |  | 0 | 0 | 0 | 0 | 0 |  | 0 | 0 | 0 | 0 | 0 |  | 0 | 0 | 0 | 0 | 0 |
| FAM1 | FAM1_8 | FAM1_5 | FAM1_4 | 2 | 2 |  | 1 | 0 | 0 | 0 | 0 |  | 1 | 0 | 0 | 0 | 0 |  | 1 | 0 | 0 | 0 | 0 |  | 1 | 0 | 0 | 0 | 0 |  | 1 | 0 | 0 | 0 | 0 |
| FAM2 | FAM2_19 | FAM2_22 | FAM2_23 | 1 | 1 |  | 0 | 0 | 0 | 0 | 0 |  | 0 | 0 | 0 | 0 | 0 |  | 0 | 0 | 0 | 0 | 0 |  | 0 | 0 | 0 | 0 | 0 |  | 0 | 0 | 0 | 0 | 0 |
| FAM2 | FAM2_23 | FAM2_41 | FAM2_42 | 2 | 2 |  | 0 | 1 | 0 | 0 | 0 |  | 0 | 1 | 0 | 0 | 0 |  | 0 | 1 | 0 | 0 | 0 |  | 0 | 1 | 0 | 0 | 0 |  | 0 | 1 | 0 | 0 | 0 |
| FAM2 | FAM2_27 | FAM2_41 | FAM2_42 | 2 | 2 |  | 0 | 1 | 0 | 0 | 0 |  | 0 | 1 | 0 | 0 | 0 |  | 0 | 1 | 0 | 0 | 0 |  | 0 | 1 | 0 | 0 | 0 |  | 0 | 1 | 0 | 0 | 0 |
| FAM2 | FAM2_33 | FAM2_41 | FAM2_42 | 2 | 2 |  | 0 | 1 | 0 | 0 | 0 |  | 0 | 1 | 0 | 0 | 0 |  | 0 | 1 | 0 | 0 | 0 |  | 0 | 1 | 0 | 0 | 0 |  | 0 | 1 | 0 | 0 | 0 |
| FAM3 | FAM3_21 | FAM3_20 | FAM3_19 | 1 | 2 |  | 0 | 0 | 1 | 0 | 0 |  | 0 | 0 | 1 | 0 | 0 |  | 0 | 0 | 1 | 0 | 0 |  | 0 | 0 | 1 | 0 | 0 |  | 0 | 0 | 1 | 0 | 0 |
| FAM3 | FAM3_28 | FAM3_34 | FAM3_33 | 2 | 2 |  | 0 | 0 | 1 | 0 | 0 |  | 0 | 0 | 1 | 0 | 0 |  | 0 | 0 | 1 | 0 | 0 |  | 0 | 0 | 1 | 0 | 0 |  | 0 | 0 | 1 | 0 | 0 |
| FAM3 | FAM3_19 | FAM3_34 | FAM3_33 | 2 | 2 |  | 0 | 0 | 1 | 0 | 0 |  | 0 | 0 | 1 | 0 | 0 |  | 0 | 0 | 1 | 0 | 0 |  | 0 | 0 | 1 | 0 | 0 |  | 0 | 0 | 1 | 0 | 0 |
| FAM3 | FAM3_27 | FAM3_34 | FAM3_33 | 1 | 1 |  | 0 | 0 | 0 | 0 | 0 |  | 0 | 0 | 0 | 0 | 0 |  | 0 | 0 | 0 | 0 | 0 |  | 0 | 0 | 0 | 0 | 0 |  | 0 | 0 | 0 | 0 | 0 |
| FAM3 | FAM3_29 | FAM3_34 | FAM3_33 | 2 | 2 |  | 0 | 0 | 1 | 0 | 0 |  | 0 | 0 | 1 | 0 | 0 |  | 0 | 0 | 1 | 0 | 0 |  | 0 | 0 | 1 | 0 | 0 |  | 0 | 0 | 1 | 0 | 0 |
| FAM4 | FAM4_1 | FAM4_47 | FAM4_48 | 1 | 2 |  | 0 | 0 | 0 | 1 | 0 |  | 0 | 0 | 0 | 1 | 0 |  | 0 | 0 | 0 | 1 | 0 |  | 0 | 0 | 0 | 1 | 0 |  | 0 | 0 | 0 | 1 | 0 |
| FAM4 | FAM4_4 | FAM4_47 | FAM4_48 | 2 | 2 |  | 0 | 0 | 0 | 1 | 0 |  | 0 | 0 | 0 | 1 | 0 |  | 0 | 0 | 0 | 1 | 0 |  | 0 | 0 | 0 | 1 | 0 |  | 0 | 0 | 0 | 1 | 0 |
| FAM4 | FAM4_34 | FAM4_47 | FAM4_48 | 1 | 2 |  | 0 | 0 | 0 | 1 | 0 |  | 0 | 0 | 0 | 1 | 0 |  | 0 | 0 | 0 | 1 | 0 |  | 0 | 0 | 0 | 1 | 0 |  | 0 | 0 | 0 | 1 | 0 |
| FAM4 | FAM4_39 | FAM4_47 | FAM4_48 | 2 | 1 |  | 0 | 0 | 0 | 0 | 0 |  | 0 | 0 | 0 | 0 | 0 |  | 0 | 0 | 0 | 0 | 0 |  | 0 | 0 | 0 | 0 | 0 |  | 0 | 0 | 0 | 0 | 0 |
| FAM4 | FAM4_42 | FAM4_47 | FAM4_48 | 1 | 1 |  | 0 | 0 | 0 | 0 | 0 |  | 0 | 0 | 0 | 0 | 0 |  | 0 | 0 | 0 | 0 | 0 |  | 0 | 0 | 0 | 0 | 0 |  | 0 | 0 | 0 | 0 | 0 |
| FAM4 | FAM4_62 | FAM4_61 | FAM4_60 | 2 | 2 |  | 0 | 0 | 0 | 1 | 0 |  | 0 | 0 | 0 | 1 | 0 |  | 0 | 0 | 0 | 1 | 0 |  | 0 | 0 | 0 | 1 | 0 |  | 0 | 0 | 0 | 1 | 0 |
| FAM5 | FAM5_1 | FAM5_48 | FAM5_49 | 1 | 2 |  | 0 | 0 | 0 | 0 | 1 |  | 0 | 0 | 0 | 0 | 1 |  | 0 | 0 | 0 | 0 | 1 |  | 0 | 0 | 0 | 0 | 1 |  | 0 | 0 | 0 | 0 | 1 |
| FAM5 | FAM5_15 | FAM5_13 | FAM5_14 | 2 | 1 |  | 0 | 0 | 0 | 0 | 0 |  | 0 | 0 | 0 | 0 | 0 |  | 0 | 0 | 0 | 0 | 0 |  | 0 | 0 | 0 | 0 | 0 |  | 0 | 0 | 0 | 0 | 0 |
| FAM5 | FAM5_22 | FAM5_48 | FAM5_49 | 2 | 2 |  | 0 | 0 | 0 | 0 | 1 |  | 0 | 0 | 0 | 0 | 1 |  | 0 | 0 | 0 | 0 | 1 |  | 0 | 0 | 0 | 0 | 1 |  | 0 | 0 | 0 | 0 | 1 |
| FAM5 | FAM5_27 | FAM5_26 | FAM5_25 | 2 | 2 |  | 0 | 0 | 0 | 0 | 1 |  | 0 | 0 | 0 | 0 | 1 |  | 0 | 0 | 0 | 0 | 1 |  | 0 | 0 | 0 | 0 | 1 |  | 0 | 0 | 0 | 0 | 1 |
| FAM5 | FAM5_33 | FAM5_48 | FAM5_49 | 2 | 2 |  | 0 | 0 | 0 | 0 | 1 |  | 0 | 0 | 0 | 0 | 1 |  | 0 | 0 | 0 | 0 | 1 |  | 0 | 0 | 0 | 0 | 1 |  | 0 | 0 | 0 | 0 | 1 |
| FAM5 | FAM5_37 | FAM5_48 | FAM5_49 | 2 | 2 |  | 0 | 0 | 0 | 0 | 1 |  | 0 | 0 | 0 | 0 | 1 |  | 0 | 0 | 0 | 0 | 1 |  | 0 | 0 | 0 | 0 | 1 |  | 0 | 0 | 0 | 0 | 1 |
| FAM5 | FAM5_45 | FAM5_48 | FAM5_49 | 2 | 1 |  | 0 | 0 | 0 | 0 | 0 |  | 0 | 0 | 0 | 0 | 0 |  | 0 | 0 | 0 | 0 | 0 |  | 0 | 0 | 0 | 0 | 0 |  | 0 | 0 | 0 | 0 | 0 |
| FAM6 | FAM6_1 | FAM6_5 | FAM6_4 | 2 | 2 |  |  |  |  |  |  |  | 0 | 0 | 0 | 0 | 0 |  | 0 | 0 | 0 | 0 | 0 |  | 0 | 0 | 0 | 0 | 0 |  | 0 | 0 | 0 | 0 | 0 |
| FAM6 | FAM6_7 | FAM6_5 | FAM6_4 | 2 | 2 |  |  |  |  |  |  |  | 0 | 0 | 0 | 0 | 0 |  | 0 | 0 | 0 | 0 | 0 |  | 0 | 0 | 0 | 0 | 0 |  | 0 | 0 | 0 | 0 | 0 |
| FAM6 | FAM6_16 | FAM6_17 | FAM6_15 | 2 | 1 |  |  |  |  |  |  |  | 0 | 0 | 0 | 0 | 0 |  | 0 | 0 | 0 | 0 | 0 |  | 0 | 0 | 0 | 0 | 0 |  | 0 | 0 | 0 | 0 | 0 |
| FAM7 | FAM7_1 | FAM7_43 | FAM7_44 | 2 | 2 |  |  |  |  |  |  |  | 0 | 0 | 0 | 0 | 0 |  | 0 | 0 | 0 | 0 | 0 |  | 0 | 0 | 0 | 0 | 0 |  | 0 | 0 | 0 | 0 | 0 |
| FAM7 | FAM7_12 | FAM7_43 | FAM7_44 | 2 | 2 |  |  |  |  |  |  |  | 0 | 0 | 0 | 0 | 0 |  | 0 | 0 | 0 | 0 | 0 |  | 0 | 0 | 0 | 0 | 0 |  | 0 | 0 | 0 | 0 | 0 |
| FAM7 | FAM7_17 | FAM7_43 | FAM7_44 | 2 | 2 |  |  |  |  |  |  |  | 0 | 0 | 0 | 0 | 0 |  | 0 | 0 | 0 | 0 | 0 |  | 0 | 0 | 0 | 0 | 0 |  | 0 | 0 | 0 | 0 | 0 |
| FAM7 | FAM7_19 | FAM7_18 | FAM7_17 | 2 | 1 |  |  |  |  |  |  |  | 0 | 0 | 0 | 0 | 0 |  | 0 | 0 | 0 | 0 | 0 |  | 0 | 0 | 0 | 0 | 0 |  | 0 | 0 | 0 | 0 | 0 |
| FAM8 | FAM8_2 | FAM8_0 | FAM8_0 | 1 | 2 |  |  |  |  |  |  |  | 0 | 0 | 0 | 0 | 0 |  | 0 | 0 | 0 | 0 | 0 |  | 0 | 0 | 0 | 0 | 0 |  | 0 | 0 | 0 | 0 | 0 |
| FAM8 | FAM8_28 | FAM8_65 | FAM8_66 | 2 | 2 |  |  |  |  |  |  |  | 0 | 0 | 0 | 0 | 0 |  | 0 | 0 | 0 | 0 | 0 |  | 0 | 0 | 0 | 0 | 0 |  | 0 | 0 | 0 | 0 | 0 |
| FAM8 | FAM8_57 | FAM8_65 | FAM8_66 | 2 | 2 |  |  |  |  |  |  |  | 0 | 0 | 0 | 0 | 0 |  | 0 | 0 | 0 | 0 | 0 |  | 0 | 0 | 0 | 0 | 0 |  | 0 | 0 | 0 | 0 | 0 |
| FAM8 | FAM8_69 | FAM8_65 | FAM8_66 | 2 | 2 |  |  |  |  |  |  |  | 0 | 0 | 0 | 0 | 0 |  | 0 | 0 | 0 | 0 | 0 |  | 0 | 0 | 0 | 0 | 0 |  | 0 | 0 | 0 | 0 | 0 |
| FAM8 | FAM8_83 | FAM8_2 | FAM8_1 | 2 | 1 |  |  |  |  |  |  |  | 0 | 0 | 0 | 0 | 0 |  | 0 | 0 | 0 | 0 | 0 |  | 0 | 0 | 0 | 0 | 0 |  | 0 | 0 | 0 | 0 | 0 |
| FAM9 | FAM9_1 | FAM9_61 | FAM9_62 | 2 | 2 |  |  |  |  |  |  |  | 0 | 0 | 0 | 0 | 0 |  | 0 | 0 | 0 | 0 | 0 |  | 0 | 0 | 0 | 0 | 0 |  | 0 | 0 | 0 | 0 | 0 |
| FAM9 | FAM9_7 | FAM9_5 | FAM9_4 | 2 | 1 |  |  |  |  |  |  |  | 0 | 0 | 0 | 0 | 0 |  | 0 | 0 | 0 | 0 | 0 |  | 0 | 0 | 0 | 0 | 0 |  | 0 | 0 | 0 | 0 | 0 |
| FAM9 | FAM9_13 | FAM9_61 | FAM9_62 | 1 | 2 |  |  |  |  |  |  |  | 0 | 0 | 0 | 0 | 0 |  | 0 | 0 | 0 | 0 | 0 |  | 0 | 0 | 0 | 0 | 0 |  | 0 | 0 | 0 | 0 | 0 |
| FAM9 | FAM9_18 | FAM9_61 | FAM9_62 | 2 | 2 |  |  |  |  |  |  |  | 0 | 0 | 0 | 0 | 0 |  | 0 | 0 | 0 | 0 | 0 |  | 0 | 0 | 0 | 0 | 0 |  | 0 | 0 | 0 | 0 | 0 |
| FAM9 | FAM9_36 | FAM9_61 | FAM9_62 | 2 | 2 |  |  |  |  |  |  |  | 0 | 0 | 0 | 0 | 0 |  | 0 | 0 | 0 | 0 | 0 |  | 0 | 0 | 0 | 0 | 0 |  | 0 | 0 | 0 | 0 | 0 |
| FAM9 | FAM9_49 | FAM9_61 | FAM9_62 | 1 | 2 |  |  |  |  |  |  |  | 0 | 0 | 0 | 0 | 0 |  | 0 | 0 | 0 | 0 | 0 |  | 0 | 0 | 0 | 0 | 0 |  | 0 | 0 | 0 | 0 | 0 |
| FAM10 | FAM10_2 | FAM10_72 | FAM10_73 | 1 | 2 |  |  |  |  |  |  |  | 0 | 0 | 0 | 0 | 0 |  | 0 | 0 | 0 | 0 | 0 |  | 0 | 0 | 0 | 0 | 0 |  | 0 | 0 | 0 | 0 | 0 |
| FAM10 | FAM10_3 | FAM10_2 | FAM10_1 | 1 | 1 |  |  |  |  |  |  |  | 0 | 0 | 0 | 0 | 0 |  | 0 | 0 | 0 | 0 | 0 |  | 0 | 0 | 0 | 0 | 0 |  | 0 | 0 | 0 | 0 | 0 |
| FAM10 | FAM10_24 | FAM10_72 | FAM10_73 | 2 | 2 |  |  |  |  |  |  |  | 0 | 0 | 0 | 0 | 0 |  | 0 | 0 | 0 | 0 | 0 |  | 0 | 0 | 0 | 0 | 0 |  | 0 | 0 | 0 | 0 | 0 |
| FAM10 | FAM10_26 | FAM10_25 | FAM10_24 | 1 | 2 |  |  |  |  |  |  |  | 0 | 0 | 0 | 0 | 0 |  | 0 | 0 | 0 | 0 | 0 |  | 0 | 0 | 0 | 0 | 0 |  | 0 | 0 | 0 | 0 | 0 |
| FAM10 | FAM10_37 | FAM10_34 | FAM10_35 | 2 | 2 |  |  |  |  |  |  |  | 0 | 0 | 0 | 0 | 0 |  | 0 | 0 | 0 | 0 | 0 |  | 0 | 0 | 0 | 0 | 0 |  | 0 | 0 | 0 | 0 | 0 |
| FAM10 | FAM10_55 | FAM10_72 | FAM10_73 | 1 | 2 |  |  |  |  |  |  |  | 0 | 0 | 0 | 0 | 0 |  | 0 | 0 | 0 | 0 | 0 |  | 0 | 0 | 0 | 0 | 0 |  | 0 | 0 | 0 | 0 | 0 |
| FAM10 | FAM10_57 | FAM10_55 | FAM10_56 | 2 | 1 |  |  |  |  |  |  |  | 0 | 0 | 0 | 0 | 0 |  | 0 | 0 | 0 | 0 | 0 |  | 0 | 0 | 0 | 0 | 0 |  | 0 | 0 | 0 | 0 | 0 |
| FAM11 | FAM11_1 | FAM11_6 | FAM11_5 | 2 | 2 |  |  |  |  |  |  |  |  |  |  |  |  |  | 0 | 0 | 0 | 0 | 0 |  | 0 | 0 | 0 | 0 | 0 |  | 0 | 0 | 0 | 0 | 0 |
| FAM11 | FAM11_2 | FAM11_6 | FAM11_5 | 2 | 2 |  |  |  |  |  |  |  |  |  |  |  |  |  | 0 | 0 | 0 | 0 | 0 |  | 0 | 0 | 0 | 0 | 0 |  | 0 | 0 | 0 | 0 | 0 |
| FAM11 | FAM11_4 | FAM11_6 | FAM11_5 | 2 | 1 |  |  |  |  |  |  |  |  |  |  |  |  |  | 0 | 0 | 0 | 0 | 0 |  | 0 | 0 | 0 | 0 | 0 |  | 0 | 0 | 0 | 0 | 0 |
| FAM12 | FAM12_1 | FAM12_57 | FAM12_58 | 2 | 2 |  |  |  |  |  |  |  |  |  |  |  |  |  | 0 | 0 | 0 | 0 | 0 |  | 0 | 0 | 0 | 0 | 0 |  | 0 | 0 | 0 | 0 | 0 |
| FAM12 | FAM12_3 | FAM12_2 | FAM12_1 | 2 | 1 |  |  |  |  |  |  |  |  |  |  |  |  |  | 0 | 0 | 0 | 0 | 0 |  | 0 | 0 | 0 | 0 | 0 |  | 0 | 0 | 0 | 0 | 0 |
| FAM12 | FAM12_29 | FAM12_57 | FAM12_58 | 1 | 2 |  |  |  |  |  |  |  |  |  |  |  |  |  | 0 | 0 | 0 | 0 | 0 |  | 0 | 0 | 0 | 0 | 0 |  | 0 | 0 | 0 | 0 | 0 |
| FAM12 | FAM12_41 | FAM12_57 | FAM12_58 | 1 | 2 |  |  |  |  |  |  |  |  |  |  |  |  |  | 0 | 0 | 0 | 0 | 0 |  | 0 | 0 | 0 | 0 | 0 |  | 0 | 0 | 0 | 0 | 0 |
| FAM13 | FAM13_1 | FAM13_68 | FAM13_69 | 2 | 2 |  |  |  |  |  |  |  |  |  |  |  |  |  | 0 | 0 | 0 | 0 | 0 |  | 0 | 0 | 0 | 0 | 0 |  | 0 | 0 | 0 | 0 | 0 |
| FAM13 | FAM13_19 | FAM13_2 | FAM13_1 | 2 | 1 |  |  |  |  |  |  |  |  |  |  |  |  |  | 0 | 0 | 0 | 0 | 0 |  | 0 | 0 | 0 | 0 | 0 |  | 0 | 0 | 0 | 0 | 0 |
| FAM13 | FAM13_49 | FAM13_68 | FAM13_69 | 2 | 2 |  |  |  |  |  |  |  |  |  |  |  |  |  | 0 | 0 | 0 | 0 | 0 |  | 0 | 0 | 0 | 0 | 0 |  | 0 | 0 | 0 | 0 | 0 |
| FAM13 | FAM13_54 | FAM13_68 | FAM13_69 | 2 | 2 |  |  |  |  |  |  |  |  |  |  |  |  |  | 0 | 0 | 0 | 0 | 0 |  | 0 | 0 | 0 | 0 | 0 |  | 0 | 0 | 0 | 0 | 0 |
| FAM13 | FAM13_59 | FAM13_68 | FAM13_69 | 1 | 2 |  |  |  |  |  |  |  |  |  |  |  |  |  | 0 | 0 | 0 | 0 | 0 |  | 0 | 0 | 0 | 0 | 0 |  | 0 | 0 | 0 | 0 | 0 |
| FAM14 | FAM14_1 | FAM14_79 | FAM14_80 | 2 | 2 |  |  |  |  |  |  |  |  |  |  |  |  |  | 0 | 0 | 0 | 0 | 0 |  | 0 | 0 | 0 | 0 | 0 |  | 0 | 0 | 0 | 0 | 0 |
| FAM14 | FAM14_25 | FAM14_79 | FAM14_80 | 2 | 2 |  |  |  |  |  |  |  |  |  |  |  |  |  | 0 | 0 | 0 | 0 | 0 |  | 0 | 0 | 0 | 0 | 0 |  | 0 | 0 | 0 | 0 | 0 |
| FAM14 | FAM14_27 | FAM14_26 | FAM14_25 | 1 | 1 |  |  |  |  |  |  |  |  |  |  |  |  |  | 0 | 0 | 0 | 0 | 0 |  | 0 | 0 | 0 | 0 | 0 |  | 0 | 0 | 0 | 0 | 0 |
| FAM14 | FAM14_44 | FAM14_79 | FAM14_80 | 2 | 2 |  |  |  |  |  |  |  |  |  |  |  |  |  | 0 | 0 | 0 | 0 | 0 |  | 0 | 0 | 0 | 0 | 0 |  | 0 | 0 | 0 | 0 | 0 |
| FAM14 | FAM14_49 | FAM14_79 | FAM14_80 | 2 | 2 |  |  |  |  |  |  |  |  |  |  |  |  |  | 0 | 0 | 0 | 0 | 0 |  | 0 | 0 | 0 | 0 | 0 |  | 0 | 0 | 0 | 0 | 0 |
| FAM14 | FAM14_71 | FAM14_79 | FAM14_80 | 2 | 2 |  |  |  |  |  |  |  |  |  |  |  |  |  | 0 | 0 | 0 | 0 | 0 |  | 0 | 0 | 0 | 0 | 0 |  | 0 | 0 | 0 | 0 | 0 |
| FAM15 | FAM15_1 | FAM15_34 | FAM15_35 | 2 | 2 |  |  |  |  |  |  |  |  |  |  |  |  |  | 0 | 0 | 0 | 0 | 0 |  | 0 | 0 | 0 | 0 | 0 |  | 0 | 0 | 0 | 0 | 0 |
| FAM15 | FAM15_26 | FAM15_34 | FAM15_35 | 2 | 2 |  |  |  |  |  |  |  |  |  |  |  |  |  | 0 | 0 | 0 | 0 | 0 |  | 0 | 0 | 0 | 0 | 0 |  | 0 | 0 | 0 | 0 | 0 |
| FAM15 | FAM15_27 | FAM15_34 | FAM15_35 | 2 | 2 |  |  |  |  |  |  |  |  |  |  |  |  |  | 0 | 0 | 0 | 0 | 0 |  | 0 | 0 | 0 | 0 | 0 |  | 0 | 0 | 0 | 0 | 0 |
| FAM15 | FAM15_31 | FAM15_34 | FAM15_35 | 2 | 2 |  |  |  |  |  |  |  |  |  |  |  |  |  | 0 | 0 | 0 | 0 | 0 |  | 0 | 0 | 0 | 0 | 0 |  | 0 | 0 | 0 | 0 | 0 |
| FAM15 | FAM15_33 | FAM15_34 | FAM15_35 | 2 | 2 |  |  |  |  |  |  |  |  |  |  |  |  |  | 0 | 0 | 0 | 0 | 0 |  | 0 | 0 | 0 | 0 | 0 |  | 0 | 0 | 0 | 0 | 0 |
| FAM15 | FAM15_115 | FAM15_110 | FAM15_25 | 1 | 2 |  |  |  |  |  |  |  |  |  |  |  |  |  | 0 | 0 | 0 | 0 | 0 |  | 0 | 0 | 0 | 0 | 0 |  | 0 | 0 | 0 | 0 | 0 |
| FAM15 | FAM15_185 | FAM15_181 | FAM15_33 | 2 | 1 |  |  |  |  |  |  |  |  |  |  |  |  |  | 0 | 0 | 0 | 0 | 0 |  | 0 | 0 | 0 | 0 | 0 |  | 0 | 0 | 0 | 0 | 0 |
| FMA16 | FAM16_1 | FAM16_6 | FAM16_5 | 1 | 2 |  |  |  |  |  |  |  |  |  |  |  |  |  |  |  |  |  |  |  | 0 | 0 | 0 | 0 | 0 |  | 0 | 0 | 0 | 0 | 0 |
| FMA16 | FAM16_2 | FAM16_6 | FAM16_5 | 2 | 2 |  |  |  |  |  |  |  |  |  |  |  |  |  |  |  |  |  |  |  | 0 | 0 | 0 | 0 | 0 |  | 0 | 0 | 0 | 0 | 0 |
| FMA16 | FAM16_3 | FAM16_6 | FAM16_5 | 2 | 1 |  |  |  |  |  |  |  |  |  |  |  |  |  |  |  |  |  |  |  | 0 | 0 | 0 | 0 | 0 |  | 0 | 0 | 0 | 0 | 0 |
| FAM17 | FAM17_1 | FAM17_30 | FAM17_31 | 2 | 2 |  |  |  |  |  |  |  |  |  |  |  |  |  |  |  |  |  |  |  | 0 | 0 | 0 | 0 | 0 |  | 0 | 0 | 0 | 0 | 0 |
| FAM17 | FAM17_12 | FAM17_2 | FAM17_1 | 2 | 1 |  |  |  |  |  |  |  |  |  |  |  |  |  |  |  |  |  |  |  | 0 | 0 | 0 | 0 | 0 |  | 0 | 0 | 0 | 0 | 0 |
| FAM17 | FAM17_20 | FAM17_30 | FAM17_31 | 1 | 2 |  |  |  |  |  |  |  |  |  |  |  |  |  |  |  |  |  |  |  | 0 | 0 | 0 | 0 | 0 |  | 0 | 0 | 0 | 0 | 0 |
| FAM17 | FAM17_23 | FAM17_30 | FAM17_31 | 2 | 2 |  |  |  |  |  |  |  |  |  |  |  |  |  |  |  |  |  |  |  | 0 | 0 | 0 | 0 | 0 |  | 0 | 0 | 0 | 0 | 0 |
| FAM18 | FAM18_13 | FAM18_8 | FAM18_9 | 2 | 2 |  |  |  |  |  |  |  |  |  |  |  |  |  |  |  |  |  |  |  | 0 | 0 | 0 | 0 | 0 |  | 0 | 0 | 0 | 0 | 0 |
| FAM18 | FAM18_22 | FAM18_8 | FAM18_9 | 2 | 2 |  |  |  |  |  |  |  |  |  |  |  |  |  |  |  |  |  |  |  | 0 | 0 | 0 | 0 | 0 |  | 0 | 0 | 0 | 0 | 0 |
| FAM18 | FAM18_25 | FAM18_8 | FAM18_9 | 2 | 2 |  |  |  |  |  |  |  |  |  |  |  |  |  |  |  |  |  |  |  | 0 | 0 | 0 | 0 | 0 |  | 0 | 0 | 0 | 0 | 0 |
| FAM18 | FAM18_28 | FAM18_8 | FAM18_9 | 2 | 2 |  |  |  |  |  |  |  |  |  |  |  |  |  |  |  |  |  |  |  | 0 | 0 | 0 | 0 | 0 |  | 0 | 0 | 0 | 0 | 0 |
| FAM18 | FAM18_45 | FAM18_44 | FAM18_16 | 1 | 1 |  |  |  |  |  |  |  |  |  |  |  |  |  |  |  |  |  |  |  | 0 | 0 | 0 | 0 | 0 |  | 0 | 0 | 0 | 0 | 0 |
| FAM19 | FAM19_1 | FAM19_72 | FAM19_73 | 2 | 2 |  |  |  |  |  |  |  |  |  |  |  |  |  |  |  |  |  |  |  | 0 | 0 | 0 | 0 | 0 |  | 0 | 0 | 0 | 0 | 0 |
| FAM19 | FAM19_3 | FAM19_2 | FAM19_1 | 2 | 1 |  |  |  |  |  |  |  |  |  |  |  |  |  |  |  |  |  |  |  | 0 | 0 | 0 | 0 | 0 |  | 0 | 0 | 0 | 0 | 0 |
| FAM19 | FAM19_13 | FAM19_72 | FAM19_73 | 1 | 2 |  |  |  |  |  |  |  |  |  |  |  |  |  |  |  |  |  |  |  | 0 | 0 | 0 | 0 | 0 |  | 0 | 0 | 0 | 0 | 0 |
| FAM19 | FAM19_17 | FAM19_72 | FAM19_73 | 2 | 2 |  |  |  |  |  |  |  |  |  |  |  |  |  |  |  |  |  |  |  | 0 | 0 | 0 | 0 | 0 |  | 0 | 0 | 0 | 0 | 0 |
| FAM19 | FAM19_25 | FAM19_72 | FAM19_73 | 1 | 2 |  |  |  |  |  |  |  |  |  |  |  |  |  |  |  |  |  |  |  | 0 | 0 | 0 | 0 | 0 |  | 0 | 0 | 0 | 0 | 0 |
| FAM19 | FAM19_35 | FAM19_72 | FAM19_73 | 2 | 2 |  |  |  |  |  |  |  |  |  |  |  |  |  |  |  |  |  |  |  | 0 | 0 | 0 | 0 | 0 |  | 0 | 0 | 0 | 0 | 0 |
| FAM20 | FAM20_1 | FAM20_4 | FAM20_3 | 2 | 2 |  |  |  |  |  |  |  |  |  |  |  |  |  |  |  |  |  |  |  | 0 | 0 | 0 | 0 | 0 |  | 0 | 0 | 0 | 0 | 0 |
| FAM20 | FAM20_2 | FAM20_0 | FAM20_0 | 1 | 1 |  |  |  |  |  |  |  |  |  |  |  |  |  |  |  |  |  |  |  | 0 | 0 | 0 | 0 | 0 |  | 0 | 0 | 0 | 0 | 0 |
| FAM20 | FAM20_10 | FAM20_4 | FAM20_3 | 1 | 2 |  |  |  |  |  |  |  |  |  |  |  |  |  |  |  |  |  |  |  | 0 | 0 | 0 | 0 | 0 |  | 0 | 0 | 0 | 0 | 0 |
| FAM20 | FAM20_12 | FAM20_4 | FAM20_3 | 2 | 2 |  |  |  |  |  |  |  |  |  |  |  |  |  |  |  |  |  |  |  | 0 | 0 | 0 | 0 | 0 |  | 0 | 0 | 0 | 0 | 0 |
| FAM20 | FAM20_14 | FAM20_4 | FAM20_3 | 1 | 1 |  |  |  |  |  |  |  |  |  |  |  |  |  |  |  |  |  |  |  | 0 | 0 | 0 | 0 | 0 |  | 0 | 0 | 0 | 0 | 0 |
| FAM20 | FAM20_15 | FAM20_4 | FAM20_3 | 2 | 2 |  |  |  |  |  |  |  |  |  |  |  |  |  |  |  |  |  |  |  | 0 | 0 | 0 | 0 | 0 |  | 0 | 0 | 0 | 0 | 0 |
| FAM20 | FAM20_16 | FAM20_4 | FAM20_3 | 1 | 2 |  |  |  |  |  |  |  |  |  |  |  |  |  |  |  |  |  |  |  | 0 | 0 | 0 | 0 | 0 |  | 0 | 0 | 0 | 0 | 0 |
| FAM21 | FAM21_1 | FAM21_6 | FAM21_5 | 1 | 2 |  |  |  |  |  |  |  |  |  |  |  |  |  |  |  |  |  |  |  |  |  |  |  |  |  | 0 | 0 | 0 | 0 | 0 |
| FAM21 | FAM21_2 | FAM21_6 | FAM21_5 | 1 | 1 |  |  |  |  |  |  |  |  |  |  |  |  |  |  |  |  |  |  |  |  |  |  |  |  |  | 0 | 0 | 0 | 0 | 0 |
| FAM21 | FAM21_4 | FAM21_6 | FAM21_5 | 2 | 2 |  |  |  |  |  |  |  |  |  |  |  |  |  |  |  |  |  |  |  |  |  |  |  |  |  | 0 | 0 | 0 | 0 | 0 |
| FAM22 | FAM22_1 | FAM22_36 | FAM22_37 | 2 | 2 |  |  |  |  |  |  |  |  |  |  |  |  |  |  |  |  |  |  |  |  |  |  |  |  |  | 0 | 0 | 0 | 0 | 0 |
| FAM22 | FAM22_4 | FAM22_36 | FAM22_37 | 2 | 2 |  |  |  |  |  |  |  |  |  |  |  |  |  |  |  |  |  |  |  |  |  |  |  |  |  | 0 | 0 | 0 | 0 | 0 |
| FAM22 | FAM22_16 | FAM22_36 | FAM22_37 | 2 | 2 |  |  |  |  |  |  |  |  |  |  |  |  |  |  |  |  |  |  |  |  |  |  |  |  |  | 0 | 0 | 0 | 0 | 0 |
| FAM22 | FAM22_29 | FAM22_36 | FAM22_37 | 2 | 1 |  |  |  |  |  |  |  |  |  |  |  |  |  |  |  |  |  |  |  |  |  |  |  |  |  | 0 | 0 | 0 | 0 | 0 |
| FAM23 | FAM23_1 | FAM23_13 | FAM23_12 | 2 | 2 |  |  |  |  |  |  |  |  |  |  |  |  |  |  |  |  |  |  |  |  |  |  |  |  |  | 0 | 0 | 0 | 0 | 0 |
| FAM23 | FAM23_2 | FAM23_13 | FAM23_12 | 2 | 2 |  |  |  |  |  |  |  |  |  |  |  |  |  |  |  |  |  |  |  |  |  |  |  |  |  | 0 | 0 | 0 | 0 | 0 |
| FAM23 | FAM23_3 | FAM23_13 | FAM23_12 | 2 | 2 |  |  |  |  |  |  |  |  |  |  |  |  |  |  |  |  |  |  |  |  |  |  |  |  |  | 0 | 0 | 0 | 0 | 0 |
| FAM23 | FAM23_4 | FAM23_13 | FAM23_12 | 1 | 1 |  |  |  |  |  |  |  |  |  |  |  |  |  |  |  |  |  |  |  |  |  |  |  |  |  | 0 | 0 | 0 | 0 | 0 |
| FAM23 | FAM23_5 | FAM23_13 | FAM23_12 | 1 | 2 |  |  |  |  |  |  |  |  |  |  |  |  |  |  |  |  |  |  |  |  |  |  |  |  |  | 0 | 0 | 0 | 0 | 0 |
| FAM24 | FAM24_3 | FAM24_2 | FAM24_1 | 2 | 1 |  |  |  |  |  |  |  |  |  |  |  |  |  |  |  |  |  |  |  |  |  |  |  |  |  | 0 | 0 | 0 | 0 | 0 |
| FAM24 | FAM24_7 | FAM24_2 | FAM24_1 | 2 | 2 |  |  |  |  |  |  |  |  |  |  |  |  |  |  |  |  |  |  |  |  |  |  |  |  |  | 0 | 0 | 0 | 0 | 0 |
| FAM24 | FAM24_37 | FAM24_59 | FAM24_60 | 1 | 2 |  |  |  |  |  |  |  |  |  |  |  |  |  |  |  |  |  |  |  |  |  |  |  |  |  | 0 | 0 | 0 | 0 | 0 |
| FAM24 | FAM24_41 | FAM24_59 | FAM24_60 | 1 | 2 |  |  |  |  |  |  |  |  |  |  |  |  |  |  |  |  |  |  |  |  |  |  |  |  |  | 0 | 0 | 0 | 0 | 0 |
| FAM24 | FAM24_43 | FAM24_41 | FAM24_42 | 2 | 1 |  |  |  |  |  |  |  |  |  |  |  |  |  |  |  |  |  |  |  |  |  |  |  |  |  | 0 | 0 | 0 | 0 | 0 |
| FAM24 | FAM24_46 | FAM24_59 | FAM24_60 | 1 | 2 |  |  |  |  |  |  |  |  |  |  |  |  |  |  |  |  |  |  |  |  |  |  |  |  |  | 0 | 0 | 0 | 0 | 0 |
| FAM25 | FAM25_1 | FAM25_10 | FAM25_11 | 2 | 2 |  |  |  |  |  |  |  |  |  |  |  |  |  |  |  |  |  |  |  |  |  |  |  |  |  | 0 | 0 | 0 | 0 | 0 |
| FAM25 | FAM25_2 | FAM25_10 | FAM25_11 | 2 | 2 |  |  |  |  |  |  |  |  |  |  |  |  |  |  |  |  |  |  |  |  |  |  |  |  |  | 0 | 0 | 0 | 0 | 0 |
| FAM25 | FAM25_3 | FAM25_10 | FAM25_11 | 2 | 2 |  |  |  |  |  |  |  |  |  |  |  |  |  |  |  |  |  |  |  |  |  |  |  |  |  | 0 | 0 | 0 | 0 | 0 |
| FAM25 | FAM25_4 | FAM25_10 | FAM25_11 | 2 | 2 |  |  |  |  |  |  |  |  |  |  |  |  |  |  |  |  |  |  |  |  |  |  |  |  |  | 0 | 0 | 0 | 0 | 0 |
| FAM25 | FAM25_5 | FAM25_10 | FAM25_11 | 1 | 1 |  |  |  |  |  |  |  |  |  |  |  |  |  |  |  |  |  |  |  |  |  |  |  |  |  | 0 | 0 | 0 | 0 | 0 |
| FAM25 | FAM25_6 | FAM25_10 | FAM25_11 | 1 | 1 |  |  |  |  |  |  |  |  |  |  |  |  |  |  |  |  |  |  |  |  |  |  |  |  |  | 0 | 0 | 0 | 0 | 0 |
| FAM25 | FAM25_99 | FAM25_10 | FAM25_11 | 2 | 2 |  |  |  |  |  |  |  |  |  |  |  |  |  |  |  |  |  |  |  |  |  |  |  |  |  | 0 | 0 | 0 | 0 | 0 |
