# Supplementary material for: Evaluation of Gene-Based Family-Based Methods to Detect Novel Genes Associated With Familial Late Onset Alzheimer Disease
Source: Front Neurosci. 2018 Apr 4;12:209. doi: 10.3389/fnins.2018.00209 (PMC5893779; doi:10.3389/fnins.2018.00209)
Supplement: Table S3 — Comparison of kinship matrices for Fam#1 and Fam#2. [file Table3.DOCX]

**Table S3.Comparison of kinship matrices for Fam#1 and Fam#2.**

| **Balding-Nichols (BN) kinship** | | | | | | | |
| --- | --- | --- | --- | --- | --- | --- | --- |
|  | **FAM1-1** | **FAM1-2** | **FAM1-3** | **FAM2-1** | **FAM2-2** | **FAM2-3** | **FAM2-4** |
| **FAM1-1** | 0.984146 | 0.55305 | 0.46238 | 0.011429 | 0.015942 | 4.07X10-6 | -0.00633 |
| **FAM1-2** | 0.55305 | 1.00101 | 0.403001 | 0.013643 | 0.002004 | -0.00179 | -0.00727 |
| **FAM1-3** | 0.46238 | 0.403001 | 1.0003 | 0.007747 | 0.016619 | 0.007253 | 0.008622 |
| **FAM2-1** | 0.011429 | 0.013643 | 0.007747 | 0.989171 | 0.462099 | 0.240107 | 0.235812 |
| **FAM2-2** | 0.015942 | 0.002004 | 0.016619 | 0.462099 | 1.00927 | 0.490067 | 0.479181 |
| **FAM2-3** | 4.07X10-6 | -0.00179 | 0.007253 | 0.240107 | 0.490067 | 1.00666 | 0.4834 |
| **FAM2-4** | -0.00633 | -0.00727 | 0.008622 | 0.235812 | 0.479181 | 0.4834 | 1.00275 |
| **Identity by State (IBS) kinship** | | | | | | | |
|  | **FAM1-1** | **FAM1-2** | **FAM1-3** | **FAM2-1** | **FAM2-2** | **FAM2-3** | **FAM2-4** |
| **FAM1-1** | 2 | 1.71273 | 1.67243 | 1.4725 | 1.44617 | 1.46686 | 1.41529 |
| **FAM1-2** | 1.71273 | 2 | 1.62385 | 1.45211 | 1.4202 | 1.44295 | 1.39458 |
| **FAM1-3** | 1.67243 | 1.62385 | 2 | 1.45202 | 1.42999 | 1.44932 | 1.40572 |
| **FAM2-1** | 1.4725 | 1.45211 | 1.45202 | 2 | 1.61465 | 1.55194 | 1.50101 |
| **FAM2-2** | 1.44617 | 1.4202 | 1.42999 | 1.61465 | 2 | 1.65616 | 1.6089 |
| **FAM2-3** | 1.46686 | 1.44295 | 1.44932 | 1.55194 | 1.65616 | 2 | 1.62884 |
| **FAM2-4** | 1.41529 | 1.39458 | 1.40572 | 1.50101 | 1.6089 | 1.62884 | 2 |
| **EPACTS (HR) kinship** | | | | | | | |
|  | **FAM1-1** | **FAM1-2** | **FAM1-3** | **FAM2-1** | **FAM2-2** | **FAM2-3** | **FAM2-4** |
| **FAM1-1** | 1 | 0.57152 | 0.47808 | 0.00427 | 0.01145 | 0.00331 | -0.00213 |
| **FAM1-2** | 0.57152 | 1 | 0.42089 | 0.00258 | -0.0024 | -0.00028 | -0.00498 |
| **FAM1-3** | 0.47808 | 0.42089 | 1 | 0.00285 | 0.01131 | 0.00665 | 0.00613 |
| **FAM2-1** | 0.00427 | 0.00258 | 0.00285 | 1 | 0.47862 | 0.25313 | 0.24482 |
| **FAM2-2** | 0.01145 | -0.0024 | 0.01131 | 0.47862 | 1 | 0.51121 | 0.49215 |
| **FAM2-3** | 0.00331 | -0.00028 | 0.00665 | 0.25313 | 0.51121 | 1 | 0.50144 |
| **FAM2-4** | -0.00213 | -0.00498 | 0.00613 | 0.24482 | 0.49215 | 0.50144 | 1 |
| **Pedigree kinship** | | | | | | | |
|  | **FAM1-1** | **FAM1-2** | **FAM1-3** | **FAM2-1** | **FAM2-2** | **FAM2-3** | **FAM2-4** |
| **FAM1-1** | 0.5 | 0.25 | 0.25 | 0 | 0 | 0 | 0 |
| **FAM1-2** | 0.25 | 0.5 | 0.25 | 0 | 0 | 0 | 0 |
| **FAM1-3** | 0.25 | 0.25 | 0.5 | 0 | 0 | 0 | 0 |
| **FAM2-1** | 0 | 0 | 0 | 0.5 | 0.25 | 0.125 | 0.125 |
| **FAM2-2** | 0 | 0 | 0 | 0.25 | 0.5 | 0.25 | 0.25 |
| **FAM2-3** | 0 | 0 | 0 | 0.125 | 0.25 | 0.5 | 0.25 |
| **FAM2-4** | 0 | 0 | 0 | 0.125 | 0.25 | 0.25 | 0.5 |
